# Looking beyond the vent to the environmental seascapes shaping deep-sea hydrothermal ecosystems

**DOI:** 10.1038/s41598-026-44060-z

**Published:** 2026-04-22

**Authors:** Abbie S. A. Chapman, Joan M. Alfaro-Lucas, Stace E. Beaulieu, Ana Colaço, Andrey Gebruk, Ivan D. Haigh, Ana Hilario, John W. Jamieson, Terue C. Kihara, Jozée Sarrazin, Verena Tunnicliffe, Amanda E. Bates

**Affiliations:** 1 Centre for Biodiversity and Environment Research, Department of Genetics, Evolution and Environment, University College London, Gower Street, London, WC1E 6BT UK; 2 Ocean and Earth Science, University of Southampton, National Oceanography Centre Southampton, University of Southampton Waterfront Campus, European Way, Southampton, SO14 3ZH UK; 3 Department of Biology, University of Victoria, Victoria, BC Canada; 4 Woods Hole Oceanographic Institution, 266 Woods Hole Road, Woods Hole, MA 02543 USA; 5 Institute of Marine Sciences - OKEANOS, University of the Azores, Rua Professor Doutor Frederico Machado 4, 9900-140 Horta, Portugal; 6 Shirshov Institute of Oceanology, Russian Academy of Sciences, Nakhimovsky Pr., 36, Moscow, Russia 117997; 7 Centre for Environmental and Marine Studies & Department of Biology, University of Aveiro, Aveiro, Portugal; 8 Department of Earth Sciences, Memorial University of Newfoundland, St. John’s, NL A1B 3X5 Canada; 9 INES Integrated Environmental Solutions UG, Südstrand 44, 26382 Wilhelmshaven, Germany; 10 Univ Brest, Ifremer, BEEP, 29280 Plouzané, France; 11 School of Earth & Ocean Sciences, University of Victoria, Victoria, BC V8P 5C2 Canada

**Keywords:** Deep-sea hydrothermal vents, Environmental variability, Geospatial, Machine learning, Open data, Seascape, Machine learning, Biodiversity, Marine biology, Marine chemistry, Physical oceanography, Geology, Ecosystem ecology

## Abstract

To manage and protect marine ecosystems, we first need a spatialised knowledge of the seascape-scale processes surrounding them. However, we lack spatially explicit understanding of how regional processes influence biological patterns in many marine systems. This is especially true of remote marine ecosystems, such as those in the deep sea. Here, we conceptualise potential seascape-scale environmental influences on deep-sea hydrothermal vent ecosystems, guided by experts and literature. We propose environmental characteristics that may shape local biodiversity patterns, such as community structure, habitat availability, and temporal stability. Next, we develop pipelines from data extraction to analysis to improve spatial data accessibility and investigate which variables can be used to draw similarities among vent fields. Finally, we group vents from different regions according to shared environmental characteristics. We show that vents that are spatially isolated and have different species pools can share similar environmental characteristics across ocean basins, including geological, oceanographic, and biological dynamics. We thus illustrate how large-scale environmental data can be used to compare seascape attributes across remote, island-like vent ecosystems. We suggest that looking beyond local scales to consider how seascapes both influence and distinguish different vent systems within a global setting is important for conservation and macroecological contexts.

**Supplementary Information:**

The online version contains supplementary material available at 10.1038/s41598-026-44060-z.

## Introduction

The deep sea is the world’s largest and most underexplored biome, but its ecosystems and their functioning are at risk from anthropogenic pressures, including direct exploitation and climate change^1,2^. Healthy deep-sea ecosystems support key functions on Earth, including nutrient cycling, food provision, and enhancing water quality^3^. Scientists and the United Nations have highlighted a need for global programs to support data collection and improve understanding of marine habitats and species, especially those in less well-studied seascapes, such as in the deep sea^4,5^. Seascapes, which are most analogous to the landscape scale in terrestrial systems, encapsulate the oceanographic and geological processes of potential influence in the wider environment surrounding habitats, defined by the ecological phenomenon of interest^6^. Whilst even today much of the deep sea and its habitats remain unmapped^7^, many wider ocean conditions have been modelled and mapped using satellite imagery.

A large body of work from terrestrial, freshwater, and marine systems links local diversity of communities to broader regional and historical processes^8^. For instance, oceanographic processes and properties such as currents, upwelling events, and water temperature can limit the distribution and abundance of marine organisms^9^. These processes can also lead to nutrient enrichment in the water column, which can promote the growth of phytoplankton and, ultimately, support higher trophic levels. Benthic conditions such as sedimentation rate, oxygen concentration, and storms can also affect the composition of local communities^10–12^. Deep-ocean systems also have links with shallower systems via nutrients, minerals, pollutants, and species dispersal^13^. Presumably, deep-sea hydrothermal vent systems are similar to other ecosystems and, thus, influenced by both local and regional processes.

Hydrothermal vents provide a compelling model system for advancing macroecological approaches to identify key regional variables that may influence local communities in the deep sea. Vent ecology has long focused on local processes or global biogeography but the links between vent ecosystems and a full range of environmental processes, from local to regional scales, remain poorly explored^8,11,14^. Despite originally being perceived as isolated systems, vents are now known to interact with the surrounding deep sea and shallower environments^12,15^. As such, vent ecosystems are fundamentally connected to, shaped by, and interact with the geological and oceanographic settings in which they persist^15,16^. Vents also harbour many endemic species with a deep historic and geographic imprint on hydrothermal-vent communities^17^, increasing the importance of regional-scale influences^8^.

Hydrothermal vents will be impacted by changing ocean conditions^18^ and are candidate ‘off-limit’ areas for commercial deep-sea mining^19,20^, yet many countries persist in seeking to explore for mining^18^. This means that, despite still discovering vents and continuing to map the ocean floor^7^, management plans will soon be needed. Static area-based management tools^21^ might not be appropriate for deep-sea vent ecosystems and, therefore, environmental characterisation will be needed to establish boundaries for dynamic area-based management, which can shift as new data on current environmental conditions and future projections are collected and modelled. Whilst data might not yet be sufficient for such management purposes, increasing availability of data and machine-learning tools for processing and analysis can now support the development of a conceptual and data processing framework to identify important regional processes which might influence the biology at the local, vent-field scale. This is especially important in a time when anthropogenic threats are escalating and there is an absence of an existing ecoregion-style framework.

Here, we first propose features of the wider seascape and ocean system expected to influence the ecology of hydrothermal vents at local, vent-field scales, drawing from expert input and available literary evidence. The vent-field scale is defined ecologically as encompassing groups of vents, or vent sites, assumed to be connected to one another by the same magma chamber^22,23^. Second, we source relevant georeferenced environmental variables from well-established data collections and document the full pipeline from data extraction to analysis so that the diverse data sources are readily accessible. Third, we select a subset of variables based on data inclusion criteria to identify seascape-scale variables which are shared or distinguish vent systems from different ocean basins. Based on our results, we advocate strongly for analyses that include the seascape for future conservation and macroecological research.

## Results

### Proposed large-scale seascape influences on deep-sea hydrothermal vent ecosystems

**Fig. 1:**
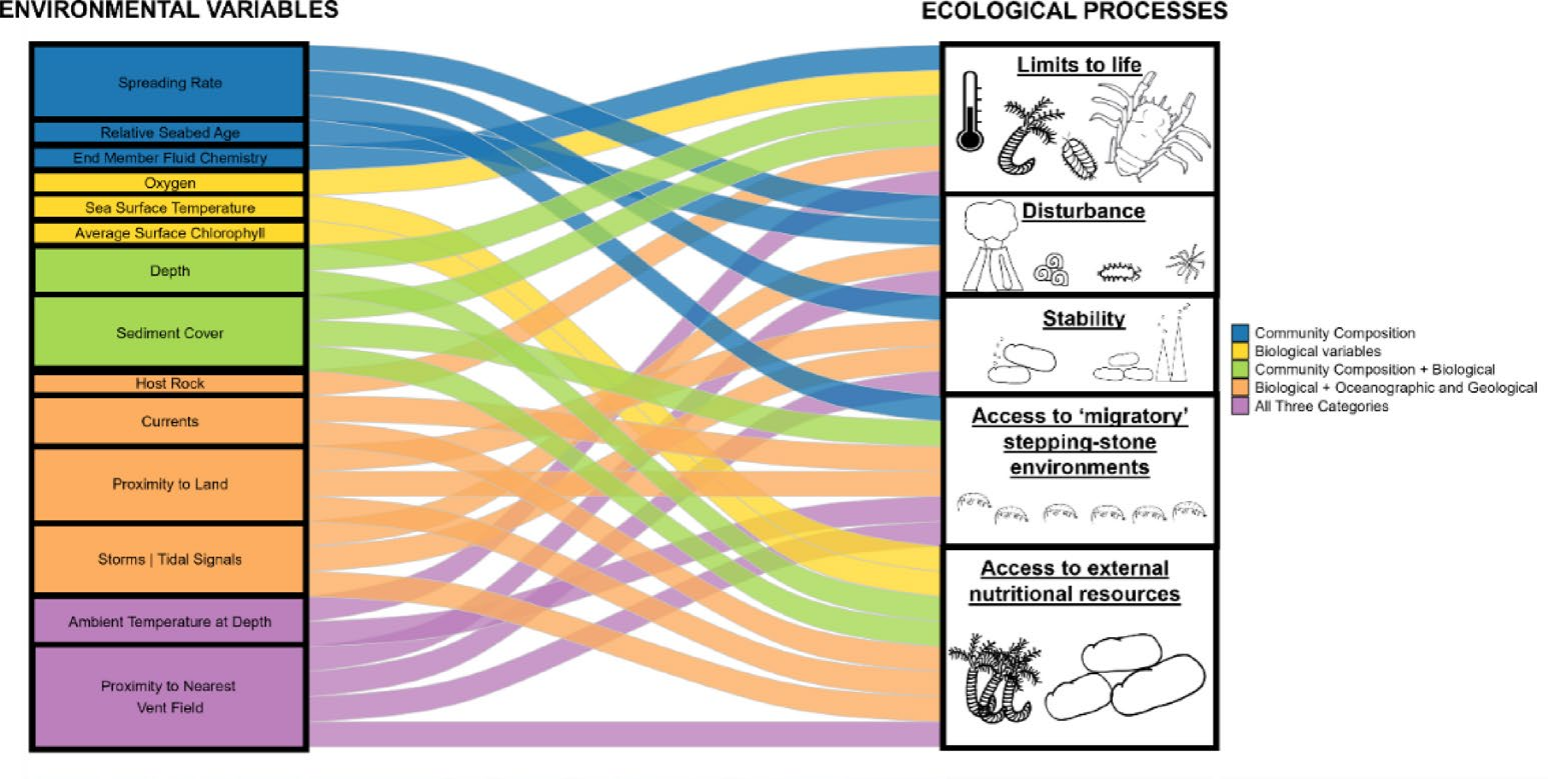
Initial conceptual framework highlighting how large-scale environmental characteristics might influence vent ecosystems, including: limits to life (i.e. environmental factors affecting which species can survive, given their physiology); access to ‘stepping stone’ environments (e.g. possible migratory pathways for mobile fauna or long-distance dispersers, and through evolutionary time for others); access to external nutritional resources; disturbance events; and factors influencing stability. An alternative visualisation of this framework (Venn diagram) is provided in Fig. S1.1 and further detail is given in Table 1. Further details on all variables are also provided in Table S1.1. The colours used for each variable outline how the environmental variables and vent-ecosystem processes are linked across scales (as shown in the key). ‘Community composition’ captures variables influencing community composition (e.g., non-vent visitors, larval survival, colonisation), ‘Biological’ are variables influencing availability of nutrients and productivity, and ‘Oceanographic and Geological’ processes affect nutrient and habitat availability, as well as environmental stability. Storms and tidal signals are grouped as they have the same connections. Note that, for example, host rock and end member fluid chemistry are not taken further in our analysis as they represent conditions at each vent site, at local, rather than seascape scales.

We present our conceptual framework, highlighting how large-scale environmental characteristics might influence deep-sea hydrothermal vent ecosystems, as an outcome in Fig. 1. The process of conceptualisation is described in detail in Methods (“Expert consensus for hypotheses on how the seascape-scale environment could influence vents”). As well as identifying links between environmental variables and ecological processes (e.g., oxygen and limits to life, and ocean currents and access to potential ‘migratory’ stepping-stone environments), we further found linked processes and variables across different scales: community composition, biological, and oceanographic and geological (Fig. 1).

### Data quantifying seascape environmental characteristics influencing vent systems

**Fig. 2:**
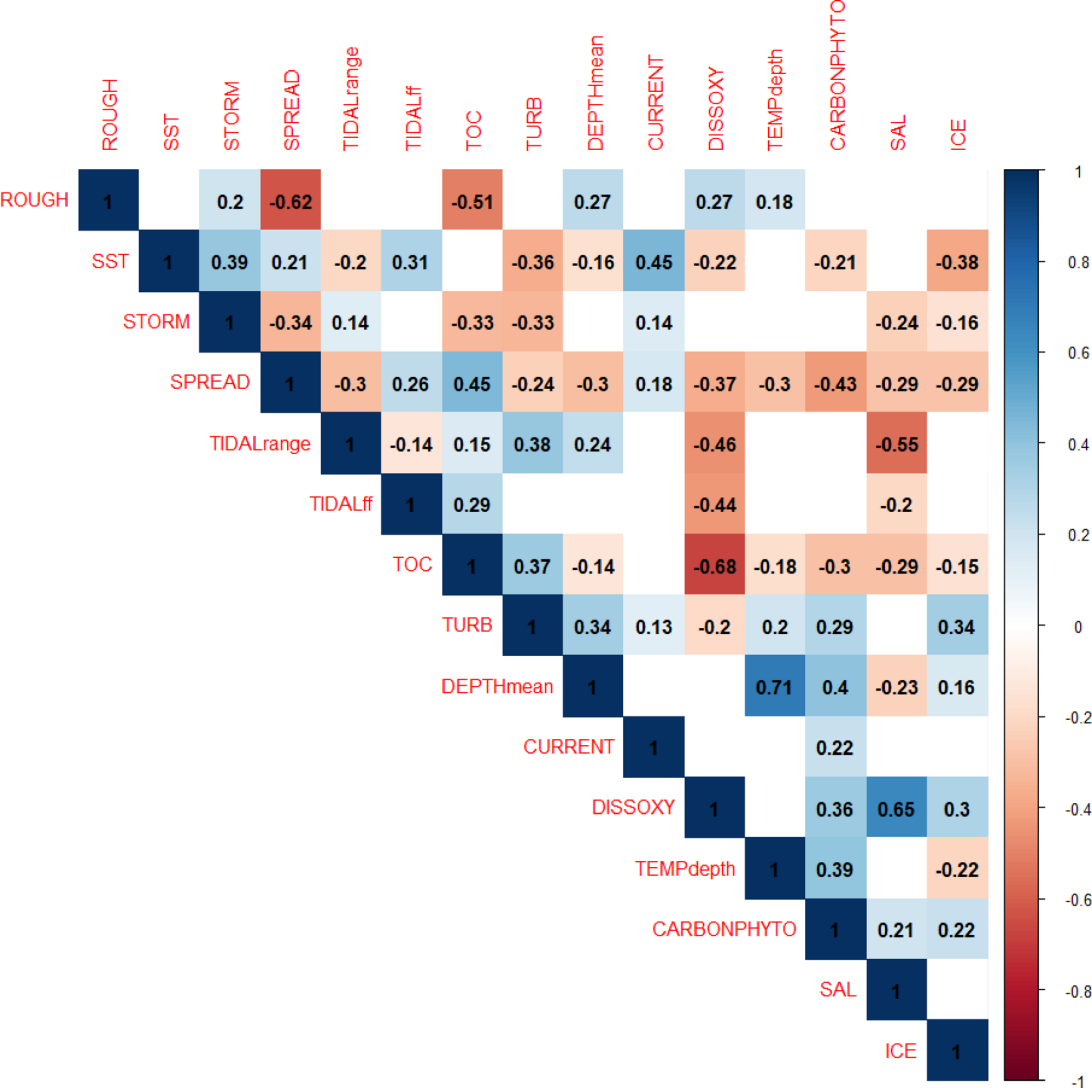
Pairwise, significant (*p* < 0.05) correlations for the fifteen environmental variables included in this part of our study. The scale is coloured according to the type of Spearman’s correlation, with blue representing positive correlation (and Spearman’s rho correlation coefficients > 0.6 considered to represent a strong correlation) and red representing negative correlation (where rho values < − 0.6 are considered to represent a strong correlation). Variable names have been shortened for presentation purposes (see Table S1.1 for abbreviations and further variable information). Only complete cases (vent fields with scores across all variables) were included in this analysis. This plot thereby represents the correlations of fifteen variables based on 166 vent fields of a possible 285 vent fields (~ 60% of the fields in the InterRidge Vents Database^24^). The correlation matrix is provided in Excel format in Dataset S3.1.

**Fig. 3:**
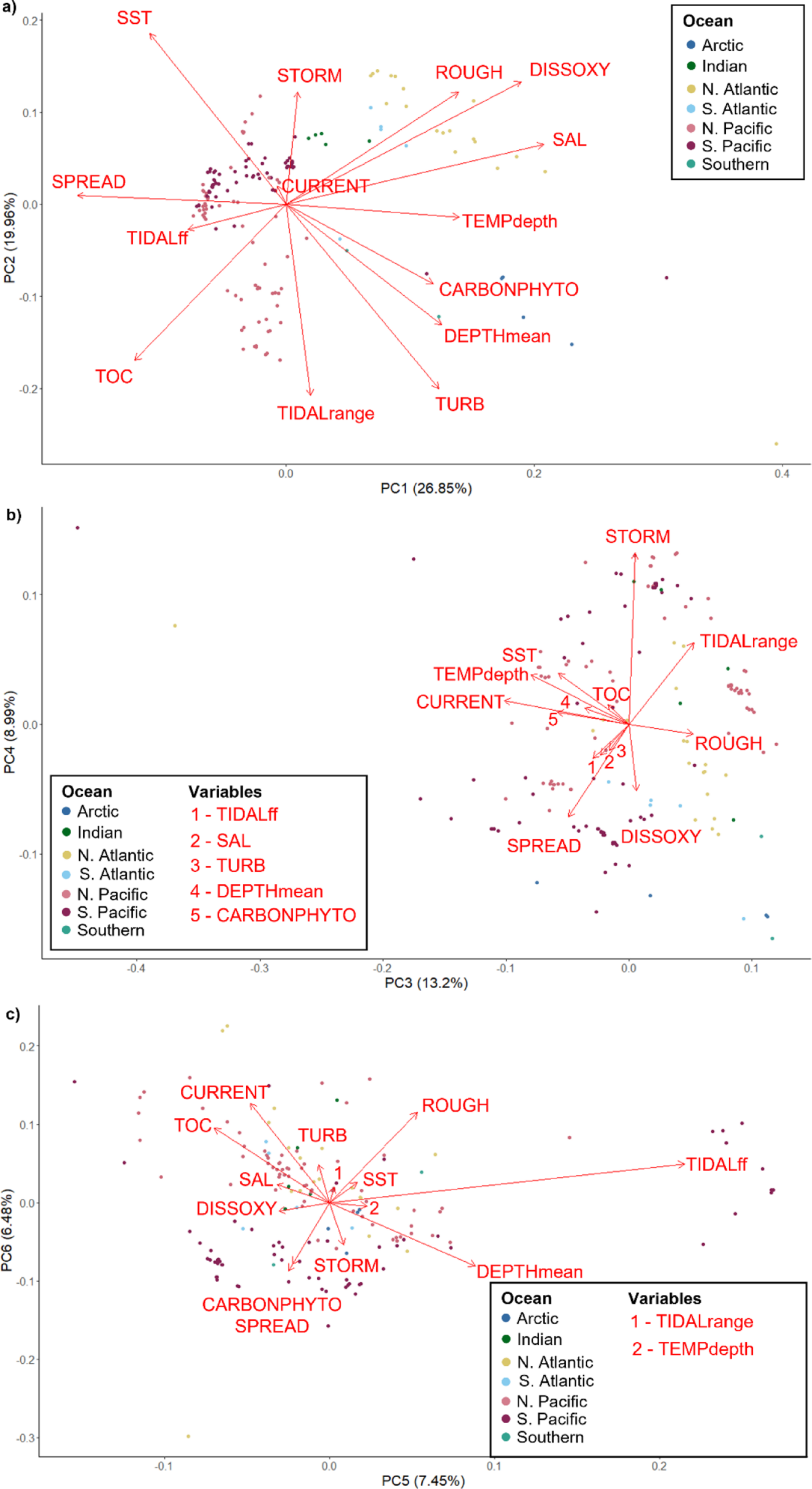
Principal component analysis (PCA) used to identify potential drivers of environmental similarity among 166 vent fields from 14 environmental variables. Principal Components (PCs) 1–5 capture 76% of the total variance, with 100% explained in 14 PCs. Panel a) shows the first two components of this PCA, together explaining 46.8% of the total variance. Panel b) represents the 3^rd^ and 4^th^ components of this PCA, capturing a further 22.2% of the total variance. Panel c) represents the 5^th^ and 6^th^ components, together explaining a further 13.9% of the total variance. Each point represents a vent field. Points are coloured according to the ocean the vent field is found within (with ‘N’ an abbreviation of North and ‘S’ an abbreviation of South). A version of these plots coloured according to vent regions is provided in Fig. S4.7 to aid interpretation for those familiar with vent biogeographic provinces. Red arrows represent the environmental variables influencing the clustering of fields, with the length of each arrow corresponding to the strength of influence (e.g., on PCs 1 and 2, average depth and phytoplankton in carbon have a similar level of influence, while currents have a much less strong influence than sea surface temperature for some fields). The arrows are labelled according to the abbreviations in Table S1.1.

We could extract complete location and environmental data for 166 active, confirmed vent fields (InterRidge Vents Database version 3.4^24^). Based on 15 variables (described in “From open data to analysis-ready data: extraction and processing of environmental variables” and summarised in Table S1.1) extracted at vent-field locations, a Spearman’s rank correlation test revealed the correlations summarised in Fig. 2. Strong, significant positive correlations (rho > 0.6, *p* < 0.05) were identified between average depth (DEPTHmean) and temperature at depth (TEMPdepth) and dissolved oxygen at depth (DISSOXY) and salinity (SAL) (Fig. 2). We found strong, significant negative correlations (rho < − 0.6, *p* < 0.05) between seafloor roughness (ROUGH) and full spreading rate (SPREAD) and total organic carbon in sediment (TOC) and dissolved oxygen at depth (DISSOXY) (Fig. 2). As ice cover (ICE) only affects seven of the 166 fields considered in our analysis (~ 4% of our dataset), we removed this variable from further analyses (Table 2). Using the remaining 14 variables (Table 2), a principal component analysis (PCA) identified potential drivers of environmental similarity among vents (Fig. 3). The majority of variance among environmental variables (> 75%) was explained by the first 5 principal components (Fig. S4.1), which can be broken down as follows. Principal Component (PC) 1 (Fig. 3) was most strongly influenced by salinity and dissolved oxygen at depth, which contributed more than 15% of the variance on this axis (Fig. S4.2). These variables separated many Atlantic fields in the North and South Mid-Atlantic Ridge (N. MAR and S. MAR) and the Terceira Rift from others (Fig. 3; Fig. S4.7). Tidal range, turbidity, and average sea-surface temperature contributed most to PC2 (Fig. 3; Fig. S4.3; Table S4.1), which we saw separating out fields in the Pacific, including the Juan de Fuca, Galapagos Rift, and Gorda and Explorer Ridge vent regions (Fig. 3; Fig. S4.7). PC3 (Fig. 3) was most influenced by current velocity and temperature (Fig. S4.4; Table S4.1), which highlighted relatively distinct fields in the Taupo Volcanic Zone in the South Pacific and the Reykjanes Ridge in the North Atlantic (Fig. 3; Fig. S4.7). Storm intensity was the dominant driver on PC4 (Fig. 3; Fig. S4.5; Table S4.1), separating conditions in more westerly parts of the South Pacific (e.g., the Lau and North Fiji Basins, among others) from South East Pacific Rise fields, for instance (Fig. 3; Fig. S4.7). Finally, tidal form factor contributed most strongly to PC5 (Fig. 3; Fig. S4.6; Table S4.1). This variable separated South Pacific fields also, defining a distinct cluster of vent fields in the Manus and Woodlark Basins (Fig. 3; Fig. S4.7).

### Grouping vents according to seascape characteristics

**Fig. 4:**
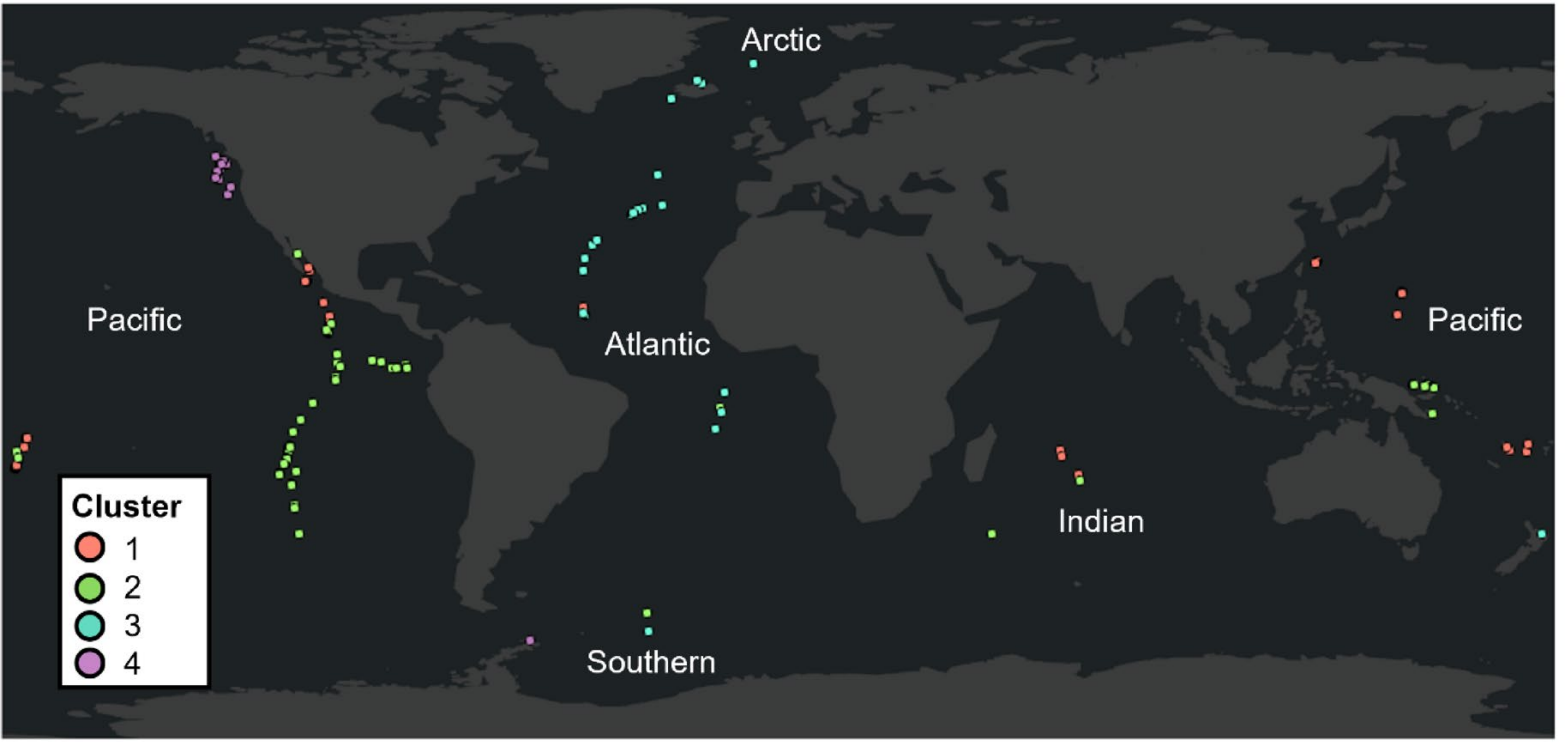
Map of the outcome of cluster analysis (partitioning around medoids, PAM, method) of environmental parameters. The vent fields (points) contained within each cluster can be identified using Dataset S5.1. The PAM cluster plots supporting this map are presented in Fig. S5.1. Oceans are labelled on this plot for reference. Colour-coding of the clusters in this figure is consistent with that used in Fig. S5.1 and Dataset S5.1. Low-confidence cluster assignments (fields with negative silhouette widths) are shown in the silhouette plot in Fig. S5.2 and Dataset S5.1.

Using the most influential variables from the PCA (n = 8) in partitioning around medoids (PAM) clustering, an unsupervised machine-learning approach, we identified four seascape-scale clusters of vent fields with shared environmental characteristics (Fig. 4; Fig. S5.1). These four clusters had an average silhouette width of 0.46 (Fig. S5.2; Dataset S5.1) and were thus sufficiently separated by the PAM clustering process to suggest the environmental variables analysed could be used to describe distinct seascapes associated with these four clusters of 46, 69, 27, and 24 vent fields. Ten vent fields remained uncertain in their cluster placement (alternative clusters are given in Dataset S5.1): CLSC A3, Ashadze, Ashadze 2, TAG, Lilliput, Baily’s Beads, White Island, Snake Pit, MAR (4 48’S), and Semyenov. CLSC A3 was placed in cluster 2 and could belong to cluster 1, Semyenov was in cluster 3 but could be in cluster 1, and the remainder were in cluster 3 but could also belong to cluster 2, as shown by negative silhouette widths and associated neighbour cluster information output from PAM clustering (Fig. S5.2; Dataset S5.1).

The first two dimensions of PAM clustering are shown in Fig. S5.1, mapped in Fig. 4, explaining nearly 60% of variance across the 166 vent fields considered in our analysis. PAM clusters 1 and 2 (salmon and green, respectively) comprised vents in the Pacific, Atlantic, and Indian Oceans, highlighting shared environmental characteristics at seascape level in different ocean basins. Contrastingly, cluster 3 was dominated by vent fields in the North Atlantic and Arctic oceans and cluster 4 the North Pacific. There were two Southern Ocean vent fields—E2 and E9—which clustered separately in the results of our PAM analysis. They were distinct from one another in PC5 of the PCA (Fig. 3), suggesting that tidal form factor, depth, and total organic carbon in sediments might vary in the area surrounding these vent fields (Fig. S4.6; Table S4.1).

## Discussion

Here, we explore large-scale environmental influences on remote deep-sea hydrothermal vents, demonstrating how open data can be leveraged to characterise the seascapes with potential to shape vent ecology. Overall, our work demonstrates that, even with several variables and a small number of groupings, we can see that vents from different regions share similar seascape-scale environmental characteristics. These groupings can inform conservation science and policy development, such as advising on how vent regions in the Southern, Atlantic, Pacific, and Indian Ocean might respond differently to mining and other stressors than regions with distinct seascapes, such as the Juan de Fuca, Gorda, and Explorer Ridge regions of the Northeast Pacific Ocean. The seascape in a conservation context is particular relevant at this time as the International Seabed Authority, in the Draft Regional Environment Management Plans for the North Atlantic (ISA/27/C/38)^25^, for example, proposes to classify active vents as sites in need of protection, but increasing numbers of countries are seeking to explore the seafloor for mining^18^. Our findings also have importance for conservation ecology. Environmental similarities among vents from different regions may filter for functional convergence and redundancy, providing resilience in cases where the selected species are tolerant of stressors, or vulnerability where this is not the case.

Some of the groupings we identify match well with biogeographic provinces familiar to vent biogeographers (e.g., Supporting Information in Alfaro-Lucas, Chapman, et al.^26^). Even so, we also find environmentally distinct clusters of vent fields, some of which are geographically separated in a way that would not have emerged in more commonly used classifications, such as those restricted by political or administrative boundaries, or within ocean basins, or focusing on species alone. The substantial number of potential seascape-scale influences on deep-sea hydrothermal vent ecosystems further emphasise a need for macroecological studies across regions for vents, rather than studying regions separately, as is common practice in vent ecology^26^. Whilst biome and ecoregion designations aim to provide a way to group habitats on the global scale for macroecological studies, our findings suggest that the spatial scale of analysis should, and can, be determined by the scales at which key larger scale regional processes influencing local habitats are operating, as proposed in a review by Swanborn et al.^6^. We show that many large-scale environmental variables differ between hydrothermal systems through longitude, latitude, and depth (x, y, and z dimensions). For instance, we find that Arctic vents are characterised by shallower depths and higher primary productivity and turbidity, which, as hypothesised in our conceptual framework, likely play an important role in community composition^27^ and on species biological traits^26^. Similarly, North Pacific vent fields are characterised by tidal ranges and storms known to affect the dynamics of their communities^12,28^. The majority of variance globally is explained in salinity, dissolved oxygen, spreading rate, and temperature at depth. Focusing on specific regions, Northeast Pacific vent fields are associated with lower salinity and sea surface temperatures, for example, which we define as limits to life, influencing productivity, dispersal, and other cross-system interactions. It is well established that spreading rates in the mid-Atlantic are slower than those in the East Pacific (as mapped), but dissolved oxygen—another limit to life, affecting nutrition, dispersal, and habitat availability—is also higher in the Mid-Atlantic Ridge regions and lowest in the Northeast Pacific. Tidal ranges are greater in the Northeast Pacific and smaller in the East Pacific and Atlantic, potentially influencing metapopulation dynamics via larval transport and exchange. Factors such as current velocity at depth, average temperatures at the surface and at depth, and storm intensity over a multi-decadal timescale also proved important for separating vent fields according to their wider environment of influence. Turbidity, for example, was greater in the Arctic and Antarctic and may thereby play a role in shaping available habitat in these regions.

The broad range of seascape-scale environmental variables available for our analysis demonstrates the potential of pooling open data for studying remote ecosystems. However, some of the data were difficult to access and assess, despite being ‘open’. The expensive and time-consuming nature of surveys in deep-sea environments^29^ hinders the rapid collection of data on habitat distributions, oceanographic currents, and other parameters needed to support species distribution modelling and other conservation network design tools^30^. For instance, more of the freely available oceanographic data are available for shallower waters, where satellite imagery can be used for measurement (e.g., temperature, chlorophyll concentration, etc.). Data on seafloor age, sediment coverage, and proximities to nearby ‘stepping-stone’ habitats such as seeps and inactive vents were not sufficient in accuracy and/or coverage to use for our analyses, so we advocate for prioritising further data collection to facilitate further seascape characterisation for the deep sea. In addition, the clusters of vents we identified by partitioning around medoids presently exclude some active, confirmed vent fields, especially as new vent fields are still being discovered. Newer vents could be added to future research using approaches such as those documented herein and our work could also be replicated for inactive vents, unconfirmed fields, and other ecosystems (e.g., habitats highlighted in the first World Ocean Assessment^16^). Furthermore, many of the variables considered here align with those used by Garcia-Soto et al.^31^ as key ocean climate change indicators (e.g., dissolved oxygen concentration, sea ice extent, and temperature). The impacts of climate change on the deep sea are only recently being evaluated^2^ and this will be an important avenue for future marine spatial planning research for deep-sea systems, as it is for other ecosystems.

‘Hidden data’ or dark data^32^, which are available but not accessible, are also an issue common to other ecosystems—marine and terrestrial^33–35^. In searching for appropriate data for this study, we came across examples of data that are technically available but have not been compiled for use in a large-scale analysis (e.g., chemistry data in Von Damm^36^). These data are likely under-used at present, as they are stored in a PDF table, or similar, and are thus not ‘analysis-ready’ without code for ‘scraping’ and additional processing steps. This prevents use for improving understanding of essential environmental variables, deemed critical by Winther et al.^37^ and Laffoley et al.^19^ for ocean management. In our work, however, we identify hidden, dark data and process them, providing supporting code to document the extraction methods, to improve their accessibility. Future work involving the mapping of hydrothermal seascapes and habitats could benefit from data tables/sets published along with manuscripts and/or in databases, and we call for these data to be made available in a standardised format with associated Digital Object Identifiers (DOIs), for old and new publications, where possible, in line with FAIR principles for scientific data management^38^.

Nevertheless, for this work, we identify and improve the accessibility of available datasets to distinguish vent fields based on large-scale environmental characteristics. Data availability limited the scope of environmental variables we could analyse (see “Methods”), but, by documenting our variable-specific methodological decisions and code, we also build and share pipelines to incorporate data which are unavailable in a standardised format. We emphasise the key step of expert discussion and conceptualisation, and thus cross-disciplinary collaboration (e.g., across ecology, geology, and physical oceanography) in our process. This ensures that: (i) relevant variables are considered; (ii) variables are only removed from analyses when appropriate; and (iii) clustering decisions are evidence-based. This has also been key in other areas of ecological research, such as the use of traits to understand functional diversity and ecosystem processes, where trait and modality selection, as well as metric parameterisation, impacts outcomes (e.g., Chapman et al.^39^; Alfaro-Lucas, Chapman et al*.*^26^). Environmental variable selection and machine-learning output threshold selection should be afforded the same diligence as trait selection in functional diversity literature. In addition to funds for data quantity, quality assurance, processing, and sharing, we propose that expert workshops should be supported, to gain maximum insights relevant for conservation and sustainable development. An exciting future lies ahead as more data are gathered and compiled, increasing capacity for quicker global-scale analyses. With this comes a need for expert-selected hypotheses and data selectivity before using machine-learning and other AI tools.

## Methods

### Expert consensus for hypotheses on how the seascape-scale environment could influence vents

We used expert knowledge during a dedicated workshop session gathering all co-authors as well as available literary evidence (Table 1) to identify the features of the wider seascape expected to be most influential on the ecology of hydrothermal vents. The term seascape captures the large-scale oceanographic and geological processes of potential influence in the environment surrounding hydrothermal vents, which is perhaps most analogous to the landscape scale in terrestrial systems.

**Table 1:** Rationale for the selection of environmental variables included in this study, with literary support.

Variable Names	Processes	Expected Ecological Impacts of Variables and Associated Processes	References
Average depth (DEPTHmean) Seafloor roughness (ROUGH) Spreading rate (SPREAD)	Limits to life Access to ‘stepping stone environments’ Access to external nutritional resources Disturbance events Environmental stability	Diversity and physiology of fauna	^11,28,34,36,40–46^
Average depth (DEPTHmean)	Limits to life Access to external nutritional resources	Interactions between systems (e.g., chemical exchanges, productivity, dispersal) and taxonomic/functional/phylogenetic similarity Relative contribution of chemosynthetic vs. photosynthetic productivity (and thus community composition, recruitment, and diversity) Pressure	^2,11,15,45,47^
Average depth (DEPTHmean) Total organic carbon in sediments (TOC)	Limits to life Access to external nutritional resources Disturbance events Environmental stability	Vent fluid temperature, composition, and deposits, affecting habitat and faunal diversity	^2,10,15,37,47^
Average depth (DEPTHmean) Phytoplankton as carbon (CARBONPHYTO) Total organic carbon in sediments (TOC)	Limits to life Access to external nutritional resources Environmental stability	Delivery of organic material / food to deep sea (≥ 2000 m) and to larvae (and thus productivity and food supply)	^2,10,11,15,37,47^
Total organic carbon in sediments (TOC)	Limits to life Access to external nutritional resources Disturbance events Environmental stability	Sediment cover (and thus habitat availability, type, and setting)	^10,15,30,37^
Tidal range (TIDALrange) Tidal form factor (TIDALff) Seafloor roughness (ROUGH) Storm intensity (STORM)	Access to ‘stepping stone environments’ Access to external nutritional resources Disturbance events Environmental stability	Species density / distribution	^12,28,36,42–44^
Tidal range (TIDALrange) Tidal form factor (TIDALff) Turbidity (TURB) Storm intensity (STORM)	Access to ‘stepping stone environments’ Access to external nutritional resources Disturbance events Environmental stability	Bottom pressure and/or mixing (including re-suspension) affects habitat availability via: Ambient temperature Chemistry Bioavailability of potentially toxic compounds	^12,28^
Current velocity (CURRENT)	Access to ‘stepping stone environments’ Access to external nutritional resources Disturbance events Environmental stability	Turbidity (including near-bottom) / particle flux (and thus food availability, sedimentation, and dispersal)	^12,15,28,43,48–50^
Tidal range (TIDALrange) Tidal form factor (TIDALff) Current velocity (CURRENT) Seafloor roughness (ROUGH)	Access to ‘stepping stone environments’ Access to external nutritional resources Disturbance events Environmental stability	Larval transport and exchange (recruitment and dispersal) and metapopulation dynamics	^12,15,27,28^
Phytoplankton as carbon (CARBONPHYTO)	Limits to life Access to external nutritional resources	Productivity and nitrogen fixation Abundance and survival of larvae (through predation) Gametogenesis	^11,42,48,51^
Sea surface temperature, salinity, sea ice, temperature at depth (SST, SAL, ICE, TEMPdepth) Dissolved oxygen (DISSOXY) Seafloor roughness (ROUGH)	Limits to life Access to ‘stepping stone environments’ Access to external nutritional resources	Water masses, ocean circulation, currents, vertical mixing, and energy dissipation, affecting chemical exchanges, productivity, dispersal, and other cross-system interactions	^11,28,36,42–45^
Spreading rate (SPREAD)	Access to ‘stepping stone environments’ Disturbance events Environmental stability	Stability (disruption of subsurface flow paths)	^28,42,46,52^

The overall conceptual framework (Fig. 1) aims to highlight how environmental characteristics influence vent systems according to the following, interconnected processes: limits to life (i.e. environmental factors affecting which species can survive, given their physiology); access to external nutritional resources; disturbance events; factors influencing stability; and access to ‘stepping stone’ environments (e.g., possible migratory pathways for mobile fauna or long-distance dispersers, and through evolutionary time for others). Here, we link the variables included in this study to these overarching ecological processes. Note that the references provided as literary support are examples and do not comprise an exhaustive list. Variable abbreviations are as defined in Table S1.1, where further details on each variable are also given.

To build the conceptual framework (Fig. 1), we grouped these features into five overarching topics influencing the ecology of hydrothermal-vent fields: (i) limits to life in vent communities; (ii) access to nutritional resources beyond the vent field; (iii) disturbance events (e.g., acute, major natural events, such as volcanic eruptions); (iv) factors influencing stability (e.g., change in environmental conditions and access to flow); and (v) access to ‘stepping-stone’ environments, influencing larval survival and metapopulation dynamics (Fig. 1; Table 1). These groupings were further classified by spatial scales into oceanographic and geological processes of influence (largest scale); drivers of nutrient and productivity availability (intermediate scale); and influencers of vent community composition (smaller scale) (Fig. 1).

Forty-one variables of potential relevance for the seascape (Table S1.1) were pooled using expert knowledge and available literature, before being reduced to 15, following filtering for appropriateness for this seascape-scale study and data limitations (see Table 2), including absence of data (e.g., a high frequency of NAs in each dataset) and lack of expert confidence in the data for our purposes (e.g., as was the case with seafloor age). Some variables (e.g., host rock) had to be excluded from further consideration and analyses because of their vent-site and vent-field-specific nature, which would not inform our understanding of the wider seascape surrounding vent fields. Similarly, proximity to nearest vent field and proximity to land (originally considered due to provision of material for possible stepping-stone environments for habitat and stability, such as wood falls via river mouths, for instance) are examples of variables not considered further because they could be biased by sampling and discovery of vent fields, which depends on research cruises and associated funding and is, thus, not consistent globally. We provide the full set of variables as a Supplementary Dataset (see S6) as a conceptual outcome of our work and flag where data gaps lie and where more and improved data are needed.

**Table 2:**
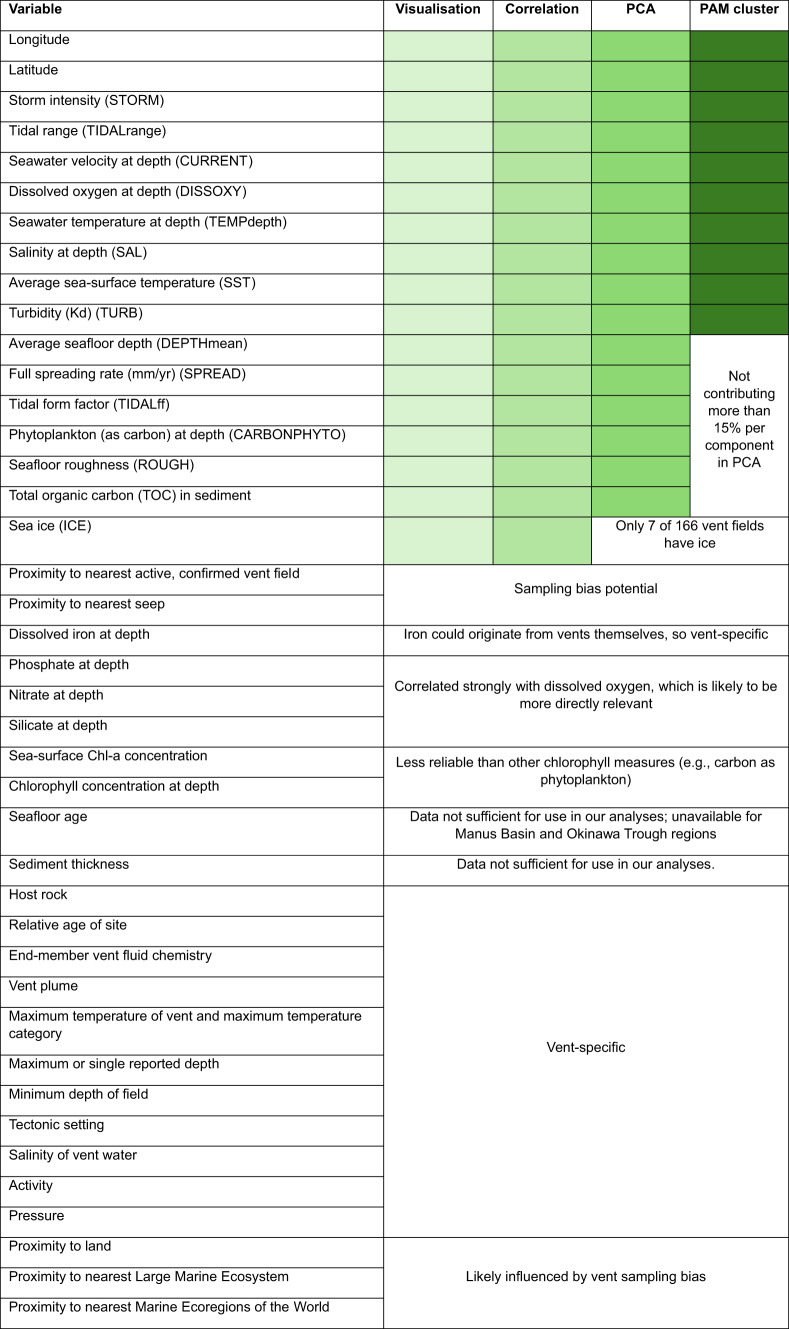
Environmental variables and the analysis stages for which they were included and excluded (with exclusion rationale).

Cells are coloured green when the variable was included in each stage of analysis (stage indicated by column header, matching descriptions in Methods) and cells with text describe the variable concerned and/or the rationale for excluding the given variable from the stage(s) of the analysis not coloured green for that variable. Visualisation refers to mapping, correlation to Spearman’s rank correlation, PCA to Principal Component Analysis, and PAM cluster to partitioning around medoids clustering (all described in Methods—“Analysis of variables of potential influence on deep-sea hydrothermal vents”).

We sourced georeferenced environmental variables fitting into the areas in our conceptual framework from well-established data collections (described in Table S1.1). The mechanisms by which we expect large-scale environmental processes to influence vent ecology are described in Fig. 1 and Table 1.

### From open data to analysis-ready data: extraction and processing of environmental variables

First, we subset data from the InterRidge Vents Database (version 3.4^24^) to identify 285 active, confirmed vent fields, of which we could extract complete location and environmental data for 166 fields (see Supplementary Materials S6—10.6084/m9.figshare.31558687). We extracted data for active, confirmed vent-field locations because these are vent fields that are actively venting fluid, and have been observed in situ, as opposed to inferred by an observation of a plume signal at the sea surface^24^. The vent-field scale is defined ecologically as encompassing groups of vents, or vent sites, assumed to be connected to one another by the same magma chamber^22,23^. We selected this scale for our analysis because vents have been unevenly sampled across the globe (preventing the use of vent-site locations for our purposes) while vent-field locations could be extracted with confidence using the InterRidge Vents Database^24^. New vent fields (e.g., Jøtul in the Arctic Ocean^53^) have been discovered since the initiation of our work, so we encourage future data users to add such vents to their analyses if appropriate. Furthermore, vent fields occupy different spatial areas, but such area/perimeter measurements are not available. Thus, we take the point locations for vent fields given in the InterRidge Vent Database^24^ and extract their environmental records. In future, should data on broader vent-field areas become available, average environmental conditions across each connected, venting area (e.g., as a polygon) could be evaluated.

For the environmental variables specifically, as with all studies dependent on large-scale data sources interpolated from satellite and in situ observations, our characterisation of seascapes is limited by data quality, resolution, and coverage^54^. Many global datasets will be lower in resolution than those compiled from local-scale measurements, especially when modelled. We justify our approach because other data are not readily available and/or comparable on the global scale and documenting data limitations for future work priorities was one of the aims of this work. Expert input was essential to ensure the data selected were suitable for the scope of our analysis.

In total, we extracted 41 environmental variables from the data sources listed in Table S1.1, including Bio-ORACLE^54,55^. The extractions highlighted a need to process the open data to make them ready for macroecological and cross-ecosystem analyses. We therefore document our approach for each variable in detail in Table 3, and provide R code in Supplementary Materials S6, as such information may also prove useful to researchers seeking similar data compilations for macroecological work in other ecosystems. Fifteen of the 41 variables could be assessed in our work (Table 2) because other variables were vent-field-specific or had inaccurate entries at the resolution required or missing data (e.g., sediment thickness and seafloor age; see Table S1.1), emphasising the importance of expert input at all stages of analysis. Examples of such data are provided in Table S1.1, including sediment thickness and seafloor age, which were identified as containing erroneous values at the scale being considered for extraction. We extracted each of the environmental variables as described in Table 3, as well as additional variables explored but identified as inappropriate for our analysis, which we make available with this manuscript for future research (see Supplementary Materials S6—10.6084/m9.figshare.31558687). More information on the original data-collection processes associated with each of the following variables and datasets is available in cited source references and metadata. Information on extractions of some of the variables considered but not included is provided in Supplementary Materials S2. Variable inclusion and exclusion criteria are documented in Table 2.

**Table 3:** Extraction and processing of environmental variables.

Variable name	Abbreviation	Description	Data source	Format	Processing
Full spreading rate	SPREAD	Spreading rate (mm/year)	InterRidge Vents Database^24^	.csv	Extracted from database directly for active, confirmed vents on mid-ocean ridges and back-arc spreading centres
Storm intensity	STORM	Tropical cyclone wind-speed buffers footprint data (‘storm intensity’—estimated maximum Saffir-Simpson cyclone category (0–5) passing over one arc-minute grid cells between 1970–2009)	Global Risk Data Platform^56^	.tif	Extracted using the ‘raster’ R package^57^
Tidal range	TIDALrange	Tidal range calculated from global tidal model	Tidal constituents from TPXO7.2 global tidal model. TPXO7.2 best fits (in a least-squares sense) the Laplace tidal equations and along‐track averaged data from the TOPEX/Poseidon and Jason altimetry missions obtained with Oregon State University Tidal Inversion Software (OTIS)	Global model to .csv	Calculated for each location following Haigh et al.^58^ using tidal constituents. Tides provided for 8 primary (M2, S2, N2, K2, K1, O1, P1, and Q1), 2 long‐period (Mf and Mm), and 3 nonlinear (M4, MS4, and MN4) harmonic constituents on a ¼ degree resolution full global grid^59,60^
Tidal form factor	TIDALff	Form factor, where: < 0.25 = semidiurnal tides, 0.25—3 = varies between diurnal and semidiurnal, and > 3 = diurnal	Tidal constituents from TPXO7.2 global tidal model	Global model to .csv	Calculated for each vent-field location following Haigh et al.^58^
Seafloor roughness	ROUGH	Roughness, mGal	earthbyte.org^61^	NetCDF	Rotated to re-project from 0–360 degree grid to -180-to-180 degree grid before extraction (R ‘raster’ package^57^)
Sea-surface temperature	SST	Decadal average (based on annual) SST (surface)	World Ocean Atlas^62^	NetCDF	Read into R using ‘raster’ package^57^
Total organic carbon in sediments	TOC	Organic carbon content in sediments (calcite)	Seiter et al.^63,64^	ASCII	Rasterised and extracted using R ‘raster’ package^57^
Turbidity	TURB	Turbidity, Kd	oceandata.sci.gsfc.nasa.gov^65^	NetCDF	Rasterised and extracted using R ‘raster’ package^57^
Depth (max)	– (In BioORACLE: ‘BO_bathymax’)	Maximum seafloor depth, 5 arc-minute resolution	BioORACLE (v0.2)^54,55^—used older version due to variable availability	Raster	Extracted using R ‘raster’ package^57^
Chlorophyll (surface)	– (In BioORACLE: ‘Average Chl-a’)	Long-term maximum mass concentration of chlorophyll in seawater at the sea surface	BioORACLE (v2.2)^54,55^, accessed using the ‘sdmpredictors’ R package^66^	Raster, 5 arcminute resolution	Extracted using R ‘raster’ package^57^
Depth (mean)	DEPTHmean (In BioORACLE: ‘BO_bathymean’)	Average seafloor depth	*As above—BioORACLE (and for all lines below)*	*As above—raster (and for all lines below)*	*As above—‘raster’ package extraction (and for all lines below)*
Chlorophyll (depth)	– (In BioORACLE: ‘BO22_chloltmax_bdmax’)	Long-term maximum mass concentration of chlorophyll in seawater at maximum depth			
Seawater velocity	CURRENT (In BioORACLE: ‘BO22_curvelltmax_bdmax’)	Long-term maximum seawater velocity at maximum depth			
Dissolved oxygen	DISSOXY (In BioORACLE: ‘BO22_dissoxltmax_bdmax’)	Long-term maximum mole concentration of dissolved oxygen in seawater at maximum depth			
Salinity	SAL (In BioORACLE: ‘BO22_salinityltmax_bdmax’)	Long-term maximum seawater salinity at the bottom at maximum bottom depth			
Iron	– (In BioORACLE: ‘BO22_ironltmax_bdmax’)	long-term maximum mole concentration of dissolved iron in seawater at maximum depth			
Phosphate	– (In BioORACLE: ‘BO22_phosphateltmax_bdmax’)	Long-term maximum mole concentration of phosphate in seawater at maximum depth			
Nitrate	– (In BioORACLE: ‘BO22_nitrateltmax_bdmax’)	Long-term maximum mole concentration of nitrate in seawater at maximum depth			
Temperature at depth	TEMPdepth (In BioORACLE: ‘BO22_templtmax_bdmax’)	Long-term maximum sea water temperature at maximum bottom depth			
Phytoplankton as carbon	CARBONPHYTO (In BioORACLE: ‘BO22_carbonphytoltmax_bdmax’)	Long-term maximum mole concentration of phytoplankton (as carbon) in seawater at maximum depth			
Silicate	– (In BioORACLE: ‘BO22_silicateltmax_bdmax’)	Long-term maximum mole concentration of silicate in seawater at maximum bottom depth			
Sea ice concentration	ICE (In BioORACLE: ‘BO22_icecoverltmax_ss’)	long-term maximum sea ice concentration (many missing values as confined to polar regions)			

The methods used to extract and process environmental variables considered in our analyses are summarised in this table, which also documents filetypes so the processes can be replicated by researchers working with other datasets of the same filetype. Some environmental data were originally processed and extracted from the World Ocean Atlas dataset^62^, but we ultimately used data freely available from the BioORACLE dataset^54,55^ to ensure that the processing was consistent among many oceanographic variables (e.g., salinity, temperature, dissolved oxygen, etc. at depth). All data were mapped using ArcMap and ArcGIS Pro GIS software^67,68^. Some of these variables, considered as local scale, were not included in our analysis, as documented in Table S1.1, where further details on all variables are given. Note that all discussions refer to characteristics of ambient seawater at depth, rather than measures of vent-fluid chemistry (e.g., we would not include temperatures emitted from vent chimneys but, instead, consider temperatures of surrounding seawater).

### Analysis of variables of potential influence on deep-sea hydrothermal vents

We first reduced each dataset to include only complete records for vent fields and variables (i.e. no ‘NA’, or missing, values) to facilitate analyses. With this complete dataset of 166 vent fields (of the 285 active, confirmed fields in the InterRidge Vent Database^24^) and 15 variables, we computed summary statistics (e.g., mean, standard deviation, range), to compare overall variability (Dataset S3.2). We mapped each variable at a global scale (Fig. S1.2) using ArcMap and ArcGIS Pro software^67,68^ (‘Visualisation’ in Table 2). We then ran a correlation test (Spearman’s rank correlation, using the ‘rcorr’ function of the ‘Hmisc’ R package, version 4.1-1^69,70^) to determine the strength of correlations among variables included in this analysis (Fig. 2; Dataset S3.1; ‘Correlation’ in Table 2). Ice cover was removed as a variable after this stage, as only 7 of 166 vent fields had ice cover values greater than zero (~ 4% of the dataset) and thus could result in these vent fields being distinct from others due to this, preventing comparison of other environmental characteristics in common, and distinct, from other fields and regions.

We used a principal component analysis (PCA) to identify which variables explain the most dissimilarity among global hydrothermal vent fields (Fig. 3; ‘PCA’ in Table 2). We conducted and presented this analysis using the ‘stats’ and ‘ggfortify’ R packages^69,71,72^. We scaled the data for cluster and PCA analyses, to standardise to a mean of zero and standard deviation of one, as the environmental variables have different units. The majority of variance (> 70%) was explained by principal components (PCs) 1–5, so we plotted variable contributions to these axes using the ‘factoextra’ package^73^ (Fig. S4.2–S4.6). The variables contributing the most (more than 15% each) to each of the first five PCs were taken forward for cluster analysis (n = 8): SAL, DISSOXY, TIDALrange, TURB, SST, CURRENT, TEMPdepth, and STORM (abbreviations as detailed in Table S1.1). We used the variable data rather than the PCs in the subsequent cluster analysis to aid interpretability as our dataset was sufficiently small for this approach.

We performed cluster analyses on the remaining eight variables to group vent fields based on environmental similarity (‘PAM cluster’ in Table 2). Cluster-based approaches are useful for identifying discrete groupings to summarise the main, simple patterns in datasets^74^. We implemented the robust^75^ ‘partitioning around medoids’ (PAM) unsupervised machine learning clustering method in R^69^ using the ‘cluster’ package (version 2.0.6^40^). We used the Gower distance dissimilarity matrix to handle our mixture of categorical (e.g. STORM) and continuous variables. We scaled our data and computed a Gower distance matrix using the ‘daisy’ function in the ‘cluster’ package before conducting the PAM analysis using the ‘pam’ function. We selected four clusters by comparing the average silhouette width of multiple runs with different ‘k’ values (numbers of clusters), seeking to maximise this value (as documented in the R script in Supplementary Material S6). Four clusters produced an average silhouette width of 0.46, as well as coherent clusters of 46, 69, 27, and 24 vent fields, with 0.52, 0.43, 0.15, and 0.75 silhouette widths, respectively (Fig. S5.2; Dataset S5.1). We produced plots of the PAM clustering (Fig. S5.1) using the ‘factoextra’ R package, before combining the cluster outputs with longitude and latitude values for each vent field to facilitate spatial mapping of the cluster outcome (Fig. 3).

## Supplementary Information

Supplementary Information.

## Data Availability

The data used in this manuscript were openly available for download, as described and cited in the methods and Supplementary Materials. The processed data are available from 10.6084/m9.figshare.31558687. We also provide a list of data sources, so others can access and use these for their ecosystems of interest. We provide the R code used to generate the results presented in this study at https://github.com/abbiesachapman/vent_seascapes. We link our extracted data (via vent-field identifiers) to the InterRidge Vents Database (10.1594/PANGAEA.917894), to ensure the data are user-friendly and can be used in conjunction with this resource.
