# Supplementary material for: Looking beyond the vent to the environmental seascapes shaping deep-sea hydrothermal ecosystems
Source: Sci Rep. 2026 Apr 22;16:20255. doi: 10.1038/s41598-026-44060-z (PMC13324470; doi:10.1038/s41598-026-44060-z)
Supplement: Supplementary file 1 — Supplementary Information. [file 41598_2026_44060_MOESM1_ESM.docx]

**SUPPLEMENTARY MATERIALS**

**S1: Conceptualising environmental characteristics of potential influence on vent fields**

**
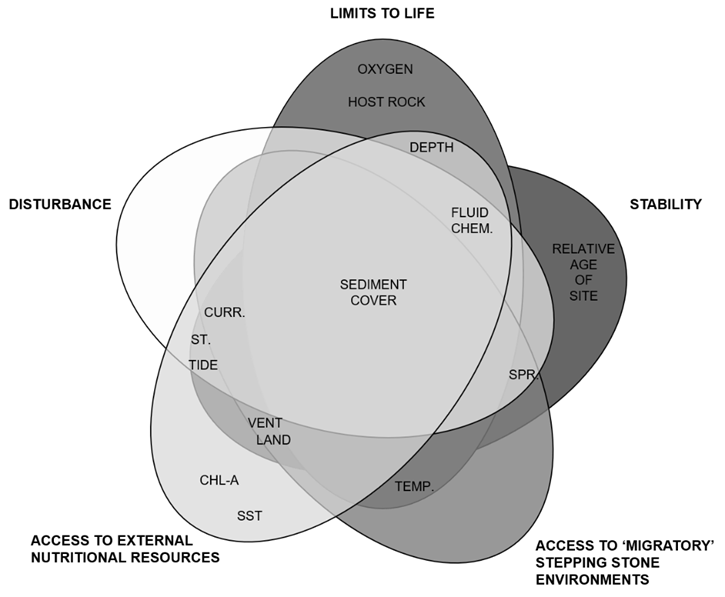
**

**Fig. S1.1: The overall conceptual framework, highlighting how environmental characteristics influence vent systems.** The framework includes: limits to life (i.e. environmental factors affecting which species can survive); access to nutritional resources; disturbance events; factors influencing stability; and access to ‘stepping stone’ environments (e.g., potential migratory pathways for mobile fauna or long-distance dispersers, and through evolutionary time for other species). This Venn diagram is presented to complement the visualization in **Fig. 1**, highlighting the overlap in both the variables and the five key areas, with some variables abbreviated for display purposes as follows: FLUID CHEM - end-member fluid chemistry; SPR - spreading rate; TEMP - ambient temperature at depth; SST - sea-surface temperature; CHL-A - average surface chlorophyll; VENT - proximity to nearest vent; LAND - proximity to land; ST - storms; TIDE - tidal signals; CURR - currents.

**Table S1.1: Environmental variables considered at various stages of our analysis, with associated source information.** Forty-one variables formed our original list, which was shortened to fifteen according to the scope of our study, as well as data gaps (i.e. high numbers of NA values) and lack of expert confidence in some datasets. After conducting a principal component analysis (PCA), eight variables were taken forward for partitioning around medoids (PAM) clustering. Grey rows emphasise these variables, included in all stages of our analysis. The ‘Abbreviation’ column refers to the short code given to each variable in some figures and tables (and variables in **Dataset S6** - <https://doi.org/10.6084/m9.figshare.31558687>). Rationale and supporting literature associated with variables is provided in **Table 1**.

| **Variable** | | **Description / Parameter(s)** | | | **Source** | | | **File type** | | **Abbreviation** |
| --- | --- | --- | --- | --- | --- | --- | --- | --- | --- | --- |
| **Longitude** | | Longitudinal location of active, confirmed vent fields. Data derived from the open literature. | | | Beaulieu and Szafrański, 2020^1^ | | | .csv | | - |
| **Latitude** | | Latitudinal location of active, confirmed vent fields. Data derived from the open literature. | | | Beaulieu and Szafrański, 2020^1^ | | | .csv | | - |
| **Seafloor depth (average)** | | Average seafloor depth (bathymetry) in metres. Note that average depth was used in analyses as deeper depths were recorded in this variable than in the maximum seafloor depth variable in some cases. BioORACLE data are compiled from satellite and in situ measured data. | | | BioORACLE v.2.2:  Tyberghein et al. (2012)^2^ and Assis et al. (2018)^3^ | | | Direct import in R using ‘sdmpredictors’ package (Bosch and Fernandez, 2021). | | DEPTHmean |
| **Proximity to nearest active, confirmed vent field** | | Minimum distance (km) between all active, confirmed vent fields (including those shallower than 200 m), as computed using geodesic distances between 285 active, confirmed vent fields in the InterRidge Vent Database (which account for the curvature of the Earth and handle fields at high latitudes and International Date Line appropriately). Location data derived from the open literature. | | | Calculated using location data available in: Beaulieu and Szafrański, 2020^1^ | | | .csv | |  |
| **Proximity to nearest seep** | | Minimum distance (km) between each active, confirmed vent field (including those shallower than 200 m) and its nearest seep, according to publicly available seep location data, compiled in 2010. Distances were computed using geodesic distances between vent fields and seeps (which account for the curvature of the Earth and handle fields near the poles and International Date Line appropriately). | | | Baker and Cuvelier, 2010, as published in German et al., 2011^4^ | | | .csv | |  |
| **Storm intensity** | | Maximum storm intensity recorded at the location of each active, confirmed vent field, on a scale from 0 to 4 (Saffir-Simpson categories), extracted from data on tropical cyclone wind-speed buffer footprint estimates from 1970-2009. | | | Raw data: IBTrACS; compilation and GIS processing: UNEP/DEWA/GRID-Europe^5^ | | | Geotiff | | STORM |
| **Full spreading rate (mm/yr)** | | Full spreading rate (in millimetres per year) for the ridge upon which each active, confirmed vent field is located. Data derived from the open literature. | | | Beaulieu and Szafrański, 2020^1^ | | | .csv | | SPREAD |
| **Host rock** | | | The host rock for a given vent. | | |  |  |  |  |  |
| **Tidal range** | | Tidal range for each location of each active, confirmed vent field (in metres), estimated using tidal constituents from the TPXO7.2 global ocean model. | | | Extracted for vent fields by I. Haigh from data as per Figure 9 of Haigh, 2017^6^ | | | .csv | | TIDALrange |
| **Tidal form factor** | | Tidal form factor for the location of each active, confirmed vent field, on a scale between 0 and 4, wherein: a score of < 0.25 indicates a semidiurnal tide; 0.25-3 suggests that it can vary between diurnal and semidiurnal; and > 3 implies a diurnal tide. The tidal form factor was calculated using tidal constituents from the TPX07.2 global tidal model. | | | Extracted for vent fields by I. Haigh from data as per Figure 9 of Haigh, 2017^6^ | | | .csv | | TIDALff |
| **Seawater velocity at depth** | | Long-term maximum seawater velocity at maximum depth (m s^-1^), compiled from data described in Tyberghein et al. (2012)^2^ and Assis et al. (2018)^3^. | | | BioORACLE v.2.2:  Tyberghein et al. (2012)^2^ and Assis et al. (2018)^3^ | | | Direct import in R using ‘sdmpredictors’ package (Bosch and Fernandez, 2021). | | CURRENT |
| **Dissolved oxygen at depth** | | Long-term maximum mole concentration of dissolved oxygen (molecular) in seawater at maximum depth (ml/l), compiled from data described in Tyberghein et al. (2012)^2^ and Assis et al. (2018)^3^. Average dissolved oxygen was also sourced but the value at depth was selected as more relevant for this study. | | | As above | | | As above | | DISSOXY |
| **Dissolved iron at depth** | | Long-term maximum mole concentration of dissolved iron in seawater at maximum depth (mmol m^-3^), compiled from data described in Tyberghein et al. (2012)^2^ and Assis et al. (2018)^3^. | | | As above | | | As above | |  |
| **Phosphate at depth** | | Long-term maximum mole concentration of phosphate in seawater at maximum depth (mmol m^-3^), compiled from data described in Tyberghein et al. (2012)^2^ and Assis et al. (2018)^3^. | | | As above | | | As above | |  |
| **Nitrate at depth** | | Long-term maximum mole concentration of nitrate in seawater at maximum depth. | | | As above | | | As above | | As above |
| **Silicate at depth** | | Long-term maximum mole concentration of silicate in seawater at maximum bottom depth. | | | As above | | | As above | | As above |
| **Seawater temperature at depth** | | Long-term maximum seawater temperature at maximum bottom depth (°C), compiled from data described in Tyberghein et al. (2012)^2^ and Assis et al. (2018)^3^. | | | As above | | | As above | | TEMPdepth |
| **Phytoplankton (as carbon) at depth** | | Long-term maximum mole concentration of phytoplankton (as carbon) in seawater at maximum depth (mmol m^-3^), compiled from data described in Tyberghein et al. (2012)^2^ and Assis et al. (2018)^3^. | | | As above | | | As above | | CARBONPHYTO |
| **Salinity at depth** | | Long-term maximum seawater salinity at the bottom at maximum bottom depth. | | | As above | | | As above | | SAL |
| **Sea-surface Chl-a concentration** | Chlorophyll-a concentration at the sea surface. | | | As above | | | As above | |  |  |
| **Chlorophyll concentration at depth** | Long-term maximum mass concentration of chlorophyll in seawater at maximum depth. | | | As above | | | As above | |  |  |
| **Sea ice** | | Long-term maximum sea ice concentration (fractional measure), compiled from data described in Tyberghein et al. (2012)^2^ and Assis et al. (2018)^3^. Average sea ice data were also collected but comprised one unique value, so were not used. | | | As above | | | As above | | ICE |
| **Average sea-surface temperature (SST)** | | Average sea-surface temperature (degrees Celsius) from the World Ocean Atlas. This was a decadal average (based on annual records). | | | World Ocean Atlas (Boyer et al., 2013) | | | NetCDF | | SST |
| **Seafloor roughness** | | Seafloor roughness for active, confirmed vent field locations in mGals (multiplied by 100 for storage purposes) and in 2-minute resolution (computed using a 100 km filter width). More information is provided in the accompanying paper stored here: http://earthbyte.org/Resources/Pdf/Whittaker_etal_seafloor_roughness_Nature200 8.pdf. | | | Whittaker et al., 2008 | | | NetCDF | | ROUGH |
| **Total organic carbon (TOC) in sediment** | | Organic carbon content of sediments (calcite). | | | Seiter et al.,2004a; Seiter et al., 2004b | | | .asc | | TOC |
| **Turbidity (Kd)** | | Turbidity measured for each active, confirmed vent field location using the downwelling diffuse attenuation coefficient based on satellite observations of downwelling spectral irradiance at 490 nm wavelength (K-d(490)) using the Visible Infrared Imaging Radiometer Suite (VIIRS). Note that as this is a satellite product it does not measure near-bottom turbidity. | | | NASA Goddard Space Flight Center, Ocean Biology Processing Group, 2014 | | | NetCDF | | TURB |
| **Seafloor age** | | Crustal age in millions of years (multiplied by 100 for storage in short integer form) for each vent field, based on data modelled and presented on a geographic grid with 2-minute resolution. More information about the origin data for this variable is provided here: https://www.ngdc.noaa.gov/mgg/ocean_age/. | | | Müller et al., 2008 | | | NetCDF | |  |
| **Sediment thickness** | | Sediment thickness estimates (average thickness over each 5-minute grid cell) in metres for the locations of each active, confirmed vent field location. Estimates modelled using maps, ocean-drilling results, and seismic reflection profiles, as described in Whittaker et al. (2013)^7^ and sources cited therein. | | | Whittaker et al., 2013^7^; Amante and Eakins, 2009^8^ | | | NetCDF | |  |
| **Relative age of site** | | This was proposed to be measured using relative sulfide deposit accumulation, relative deposit size, and/or surface area. | | |  | | |  | |  |
| **End-member vent fluid chemistry** | | This was proposed as the fundamental characteristics of this. | | |  | | |  | |  |
| **Vent plume** | | Hydrogen, particulate intensity, manganese, oxidative reduction potential. | | |  | | |  | |  |
| **Maximum temperature of vent and maximum temperature category** | | Maximum temperature and temperature category of a given vent. | | |  | | |  | |  |
| **Maximum or single reported depth** | | Maximum or single reported depth of a given vent. Note that maximum seafloor depth was also extracted from BioORACLE but was removed as the average depth had deeper values in the dataset. | | |  | | |  | |  |
| **Minimum depth of field** | | Minimum reported depth in a vent field. | | |  | | |  | |  |
| **Tectonic setting** | | Tectonic setting of a vent/vent field. | | |  | | |  | |  |
| **Salinity of vent water** | | Salinity of vent fluid specifically. | | |  | | |  | |  |
| **Activity** | | Venting activity (e.g., active, inactive, waning). | | |  | | |  | |  |
| **Pressure** | | Water pressure at the vent location. | | |  | | |  | |  |
| **Proximity to land** | | Proximity to nearest land feature. | | |  | | |  | |  |
| **Proximity to nearest Large Marine Ecosystem** | | Proximity to nearest Large Marine Ecosystem (LME). | | |  | | |  | |  |
| **Proximity to nearest Marine Ecoregions of the World** | | Proximity to nearest Marine Ecoregions of the World (MEOW). | | |  | | |  | |  |

**Fig. S1.2 (overleaf): Maps documenting spatial variability in the environmental characteristics included in our analysis at the locations of active, confirmed vent fields on a global scale.** Variables include: a) CARBONPHYTO, b) CURRENT, c) DEPTHmean, d) ICE, e) ROUGH, f) SAL, g) SPREAD, h) TOC, i) DISSOXY, j) SST, k) STORM, l) TIDALff, m) TIDALrange, n) TURB, and o) TEMPdepth (abbreviations in **Table S1.1**). Panel p) is a map of the vent fields analysed, with text labels providing a rough depiction of vent region locations and names (note that MAR is an abbreviation of Mid-Atlantic Ridge and EPR the East Pacific Rise). Vent fields named in italics in panel p) are those at the edges of PAM clusters and are provided as examples only.


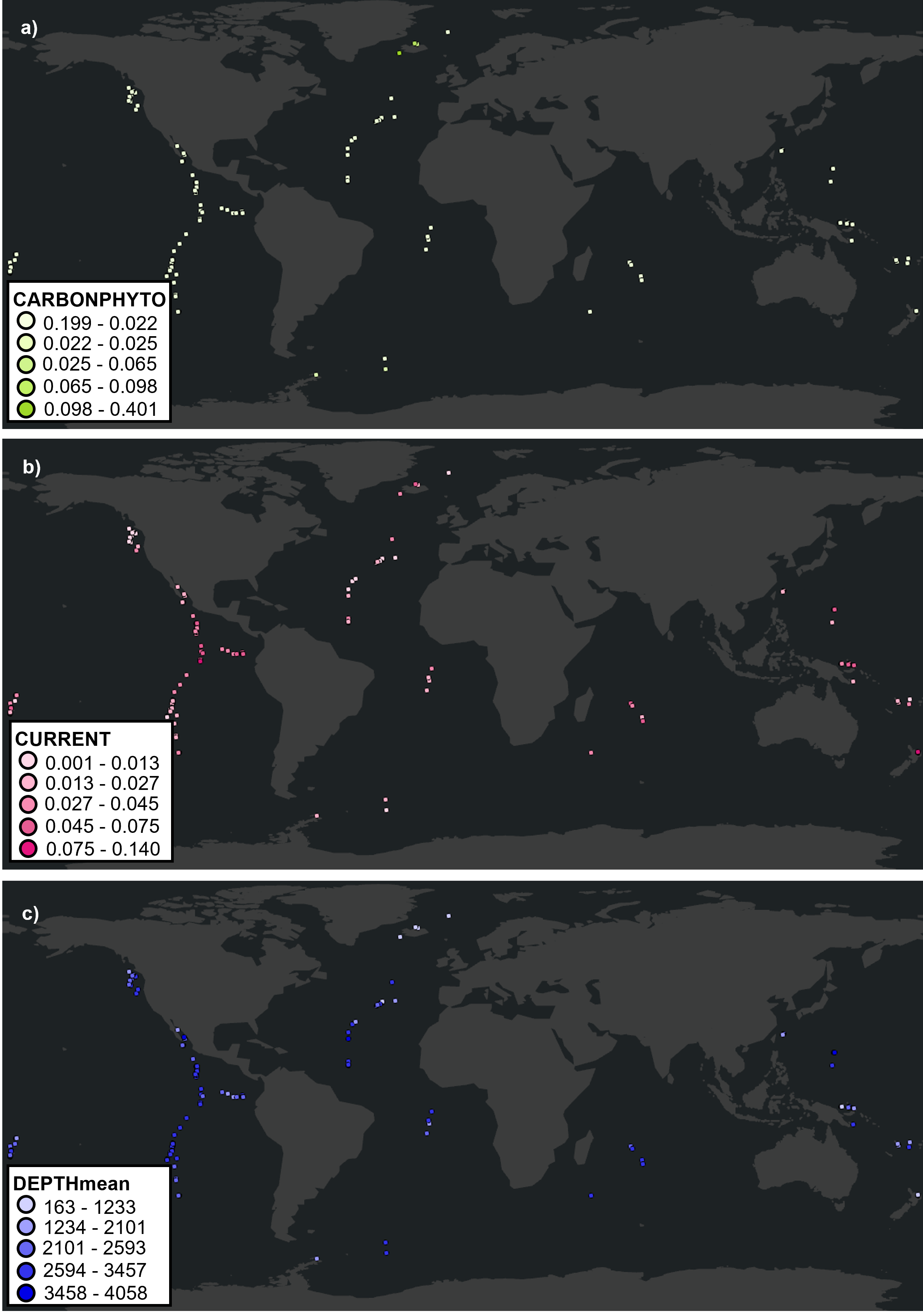

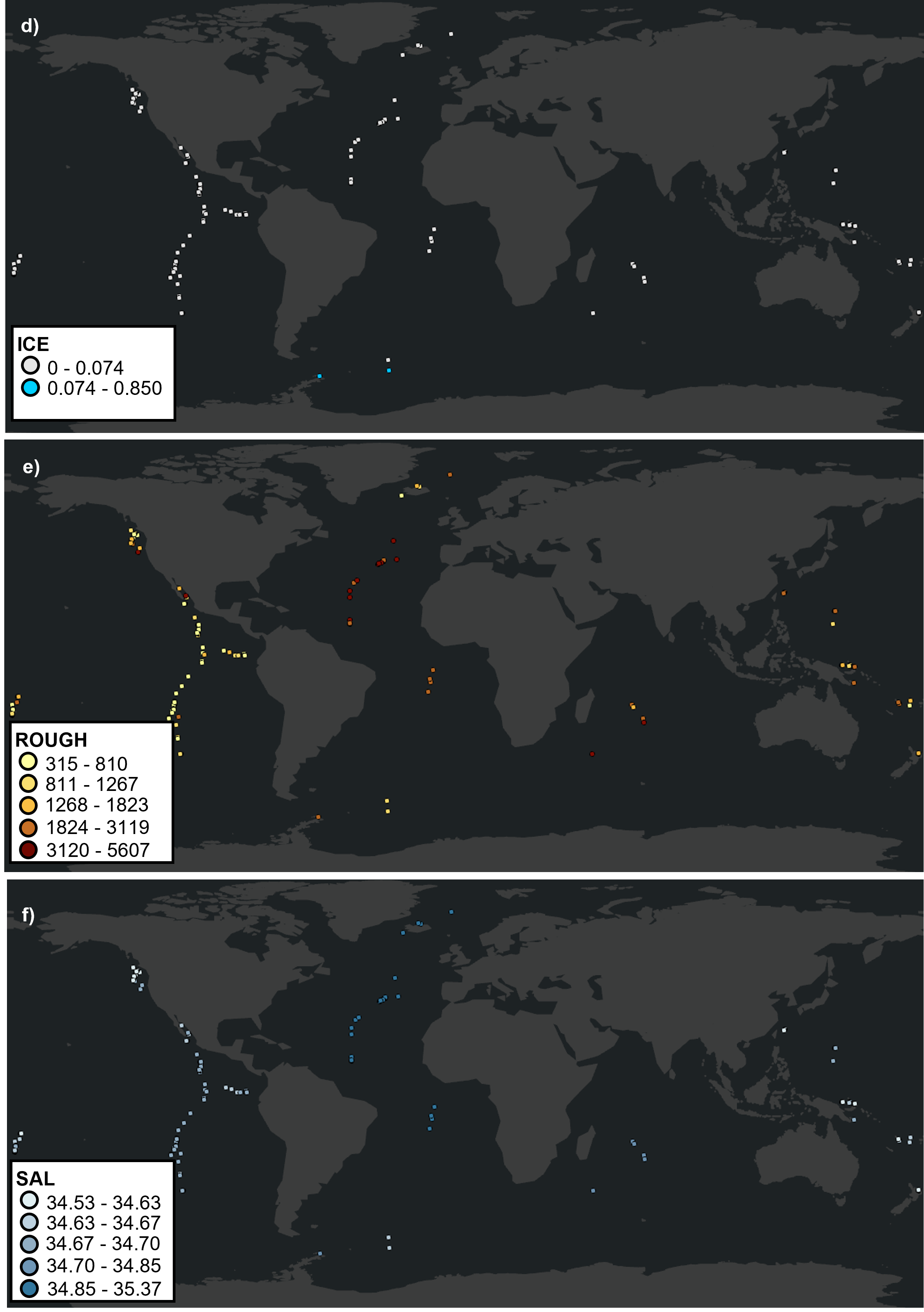

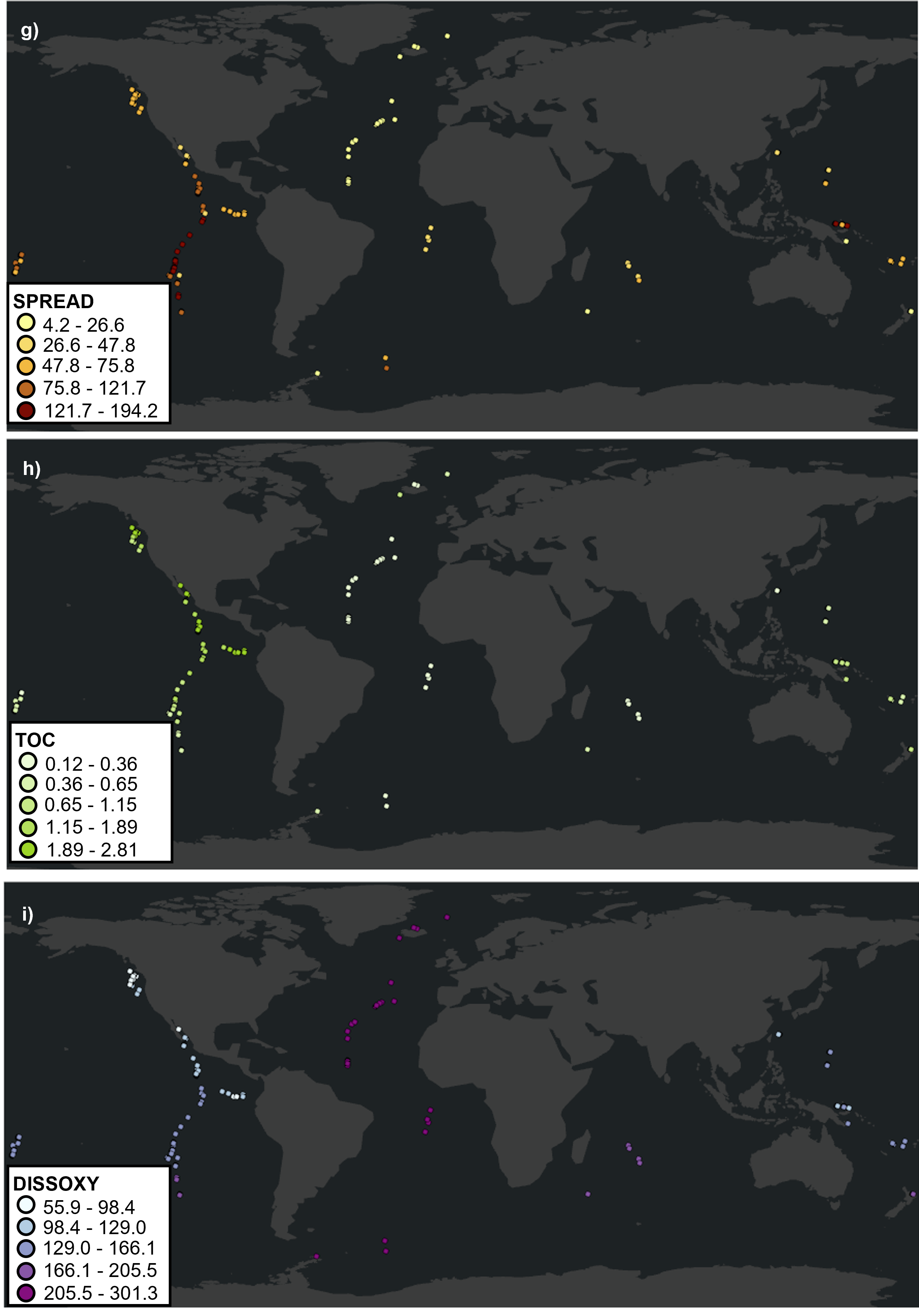

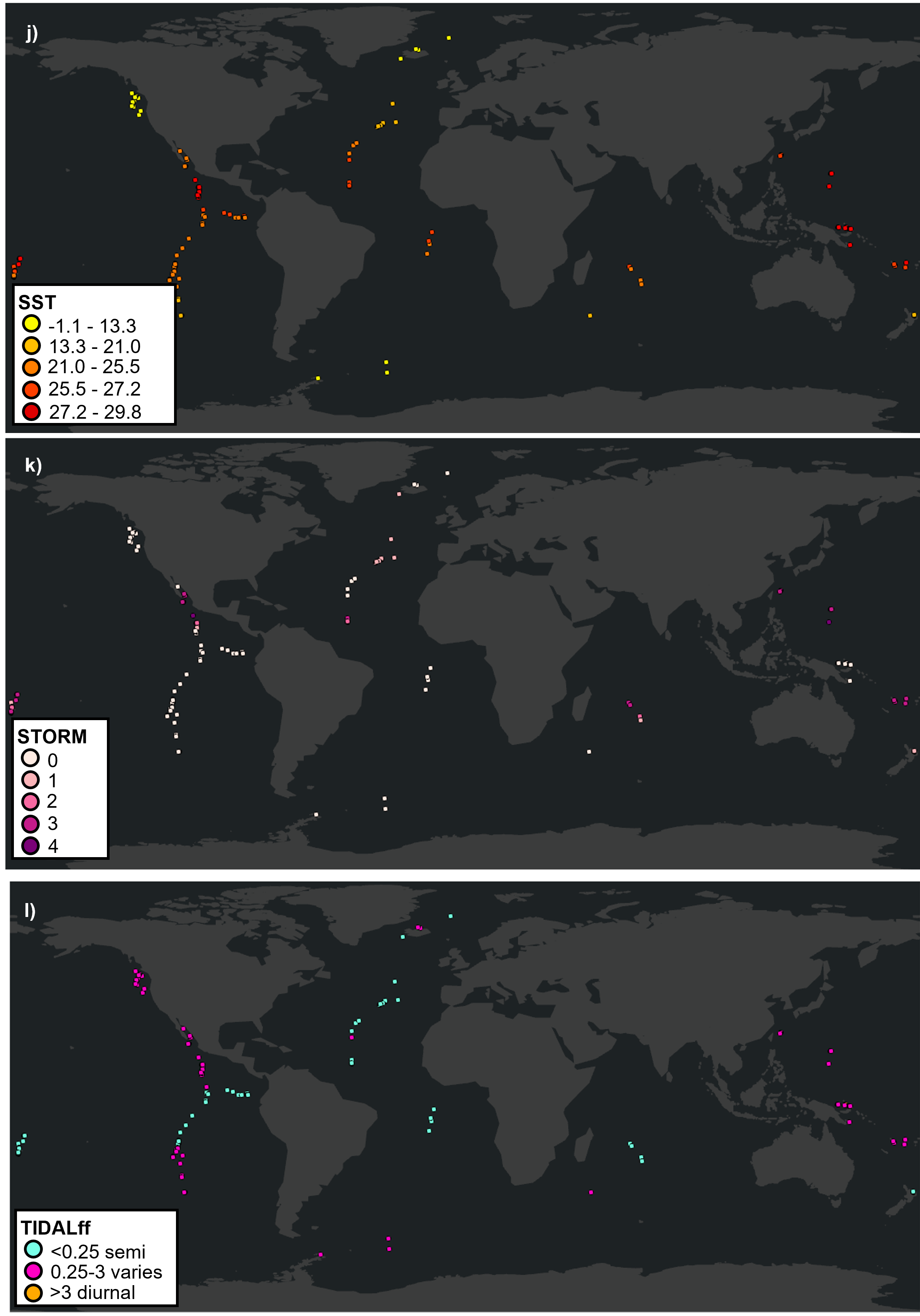

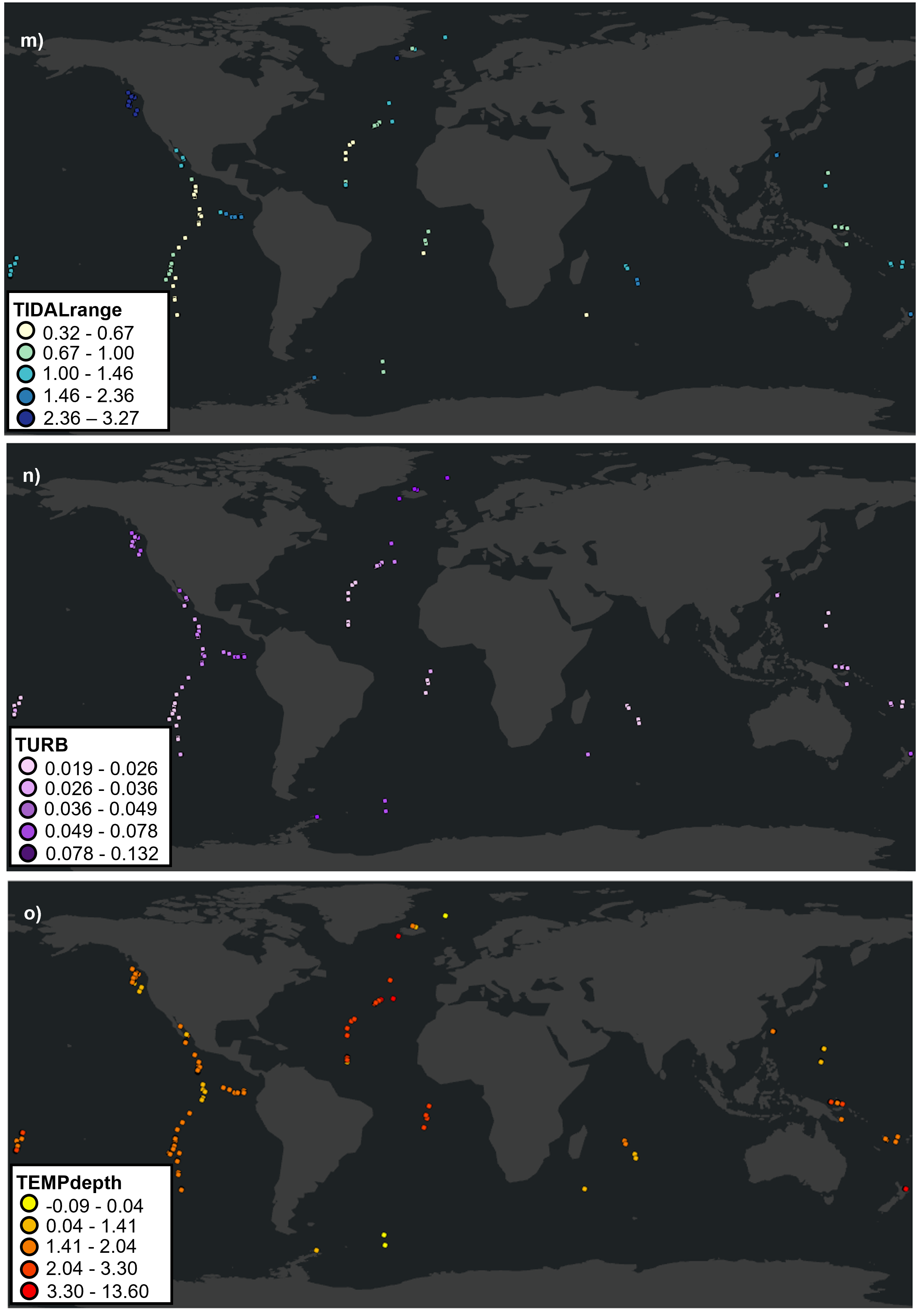

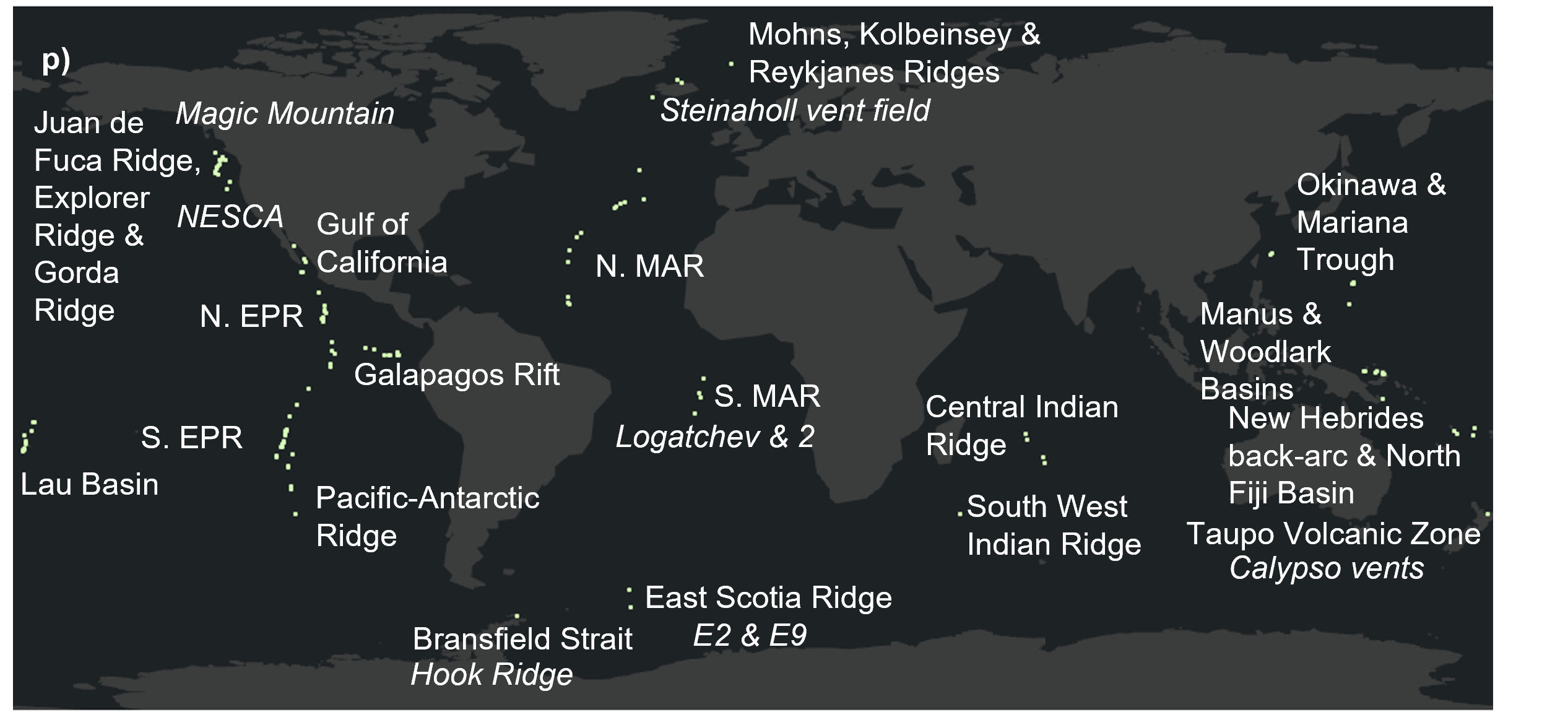


**S2: Further extraction methodology information for variables not included in analyses but of potential interest to vent ecologists**

S2.1 Proximity to nearest known active, confirmed vent field

We computed these proximities using the locations of the full set of 285 active, confirmed vent fields (longitude and latitude) accessed from the InterRidge Vents Database^41^, which we read into ArcMap GIS software (version 10.3^45^) and projected into the WGS 1984 geographic coordinate system, before exporting as a shapefile for processing. We computed the geodesic distance between each vent field and its nearest vent field using the ‘Near’ function within the ‘Proximity’ toolset of the ‘Analysis Tools’ in ArcToolbox^45^. We then exported the results to the vent-field attribute table, which we converted to an Excel file using the ‘Table to Excel’ function in ‘Conversion Tools’, so it could be read into R^46^, and converted from metres to kilometres, before analysis. Note that ‘PROXvent_all’ is also a variable provided in the dataset associated with this manuscript, calculated using the method described here but for all vent fields in the InterRidge Vents Database^41^. This variable represents the proximity of each vent field to any other vent field (i.e. including those that are unconfirmed and/or inactive). PROXvent_all was not included in our analyses due to uncertainty introduced by sampling bias (i.e. parts of a ridge might be unexplored but have many vent fields that are simply not yet included in the InterRidge Vents Database) but this variable is provided as data-users may wish to use it in future research.

S2.2 Proximity to nearest seep

Location data for seeps were provided by the ChEss project^47^. We read these data into ArcMap GIS software^45^ with InterRidge Vents Database vent field locations^41^ and assigned both datasets the geographic coordinate system WGS 1984, before exporting as shapefiles for processing. We computed the geodesic distance between each vent field and its nearest seep using the ‘Near’ function within the ‘Proximity’ toolset of the ‘Analysis Tools’ in ArcToolbox^45^. We added the results to the attribute table for the vent-field location data, before converting this to an Excel file using the ‘Table to Excel’ function in ‘Conversion Tools’ so it could be read into R^46^, and converted from metres to kilometres, before analysis. We note that proximity to nearest seep is limited by the lack of a more recent compilation of seeps discovered since 2010. Proximity may increase for some vent fields with the inclusion of more recent data; we recommend this as a priority for future work.

S2.3 Sediment thickness

Gridded global sediment thickness data (5 arc-minute resolution) were available via ngdc.noaa.gov^51^. Using the ‘raster’ package in R^46,49^, we: i) imported the data as raster data using the ‘raster’ function; ii) re-projected to the required extent (-180 to 180 degrees) using the ‘rotate’ function; and then iii) extracted for the InterRidge vent field locations using function ‘extract’. A binary classification (sedimented vs. non-sedimented) based on expert opinion could be used in future, should all fields be suitable for representation in such a way, but this may not represent the broader seascape, hence our decision to use open data on seafloor sediment thickness for the analysis we present here.

**S3: Correlations between environmental variables**

**Dataset S3.1:** The Excel file ‘correlation_matrix.csv’ is provided on figshare (<https://doi.org/10.6084/m9.figshare.31558687>) for reference, showing the Pearson’s correlation coefficient (‘Correlation’ column) and p-value for each pair of variables.

**Dataset S3.2:** The Excel file “summarystatistics.csv” is provided on figshare (<https://doi.org/10.6084/m9.figshare.31558687>) for reference, providing summary statistics for the 15 variables analysed across 166 vent fields.

**S4: Supplementary Principal Component Analysis (PCA) outcomes**


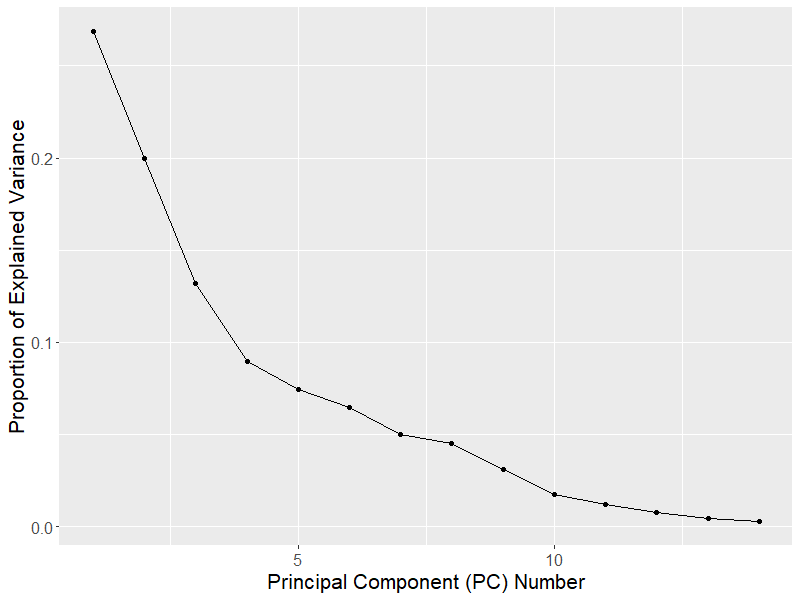


**Fig. S4.1: Screeplot from principal component analysis (PCA)**. This is used to identify an ‘elbow’ point, which gives an indication of a good number of clusters. There are multiple options for this dataset, but 5 Principal Components (PCs) explain the majority of variance and form an elbow point in this plot.


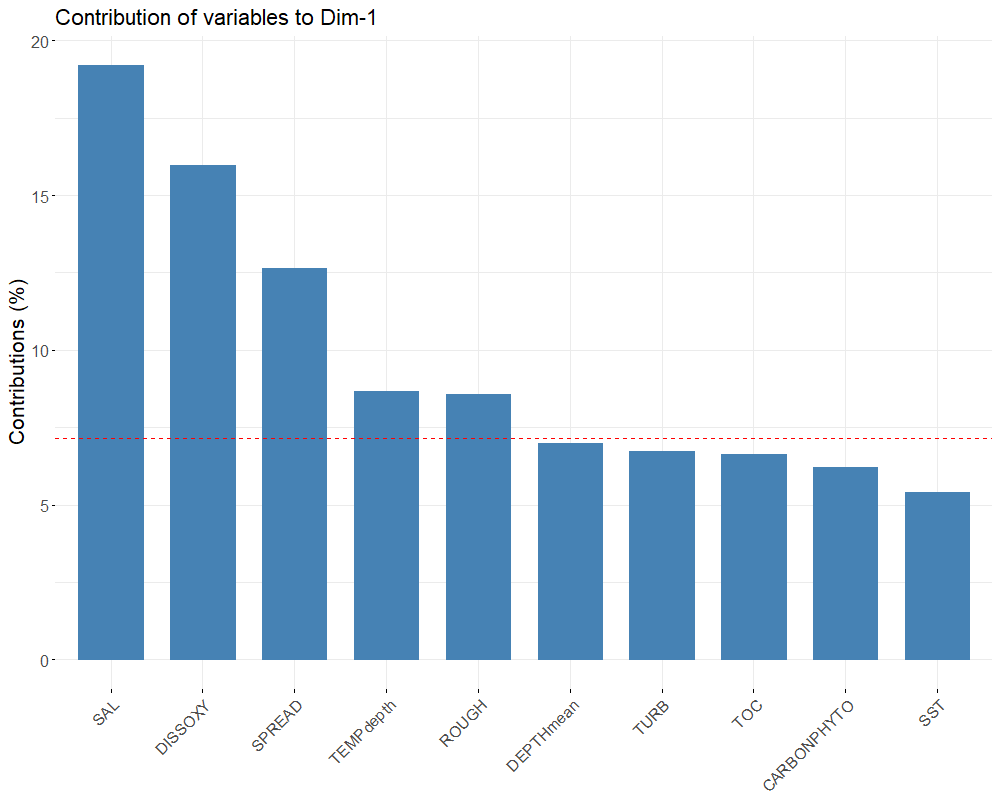


**Fig. S4.2: Plot outlining the relative contributions of different variables to the first dimension of the PCA (PC1 or Dim1)**. The red line is used as a reference, but contributions above 15% were considered the most important in our analysis. Here, salinity (SAL) and dissolved oxygen (DISSOXY) meet this criterion. Abbreviations are as provided in **Table S1.1**.


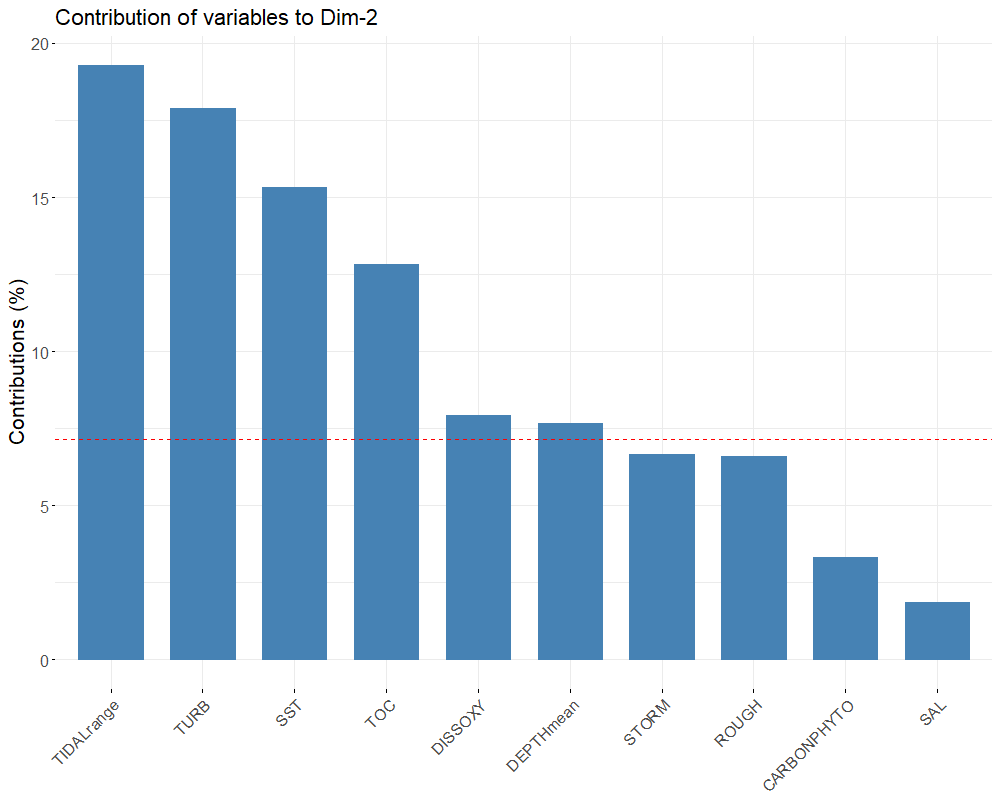


**Fig. S4.3: Plot outlining the relative contributions of different variables to the second dimension of the PCA (PC2 or Dim2)**. The red line is used as a reference, but contributions above 15% were considered the most important in our analysis. Here, tidal range (TIDALrange), turbidity (TURB), and sea-surface temperature (SST) meet this criterion. Abbreviations are as provided in **Table S1.1**.


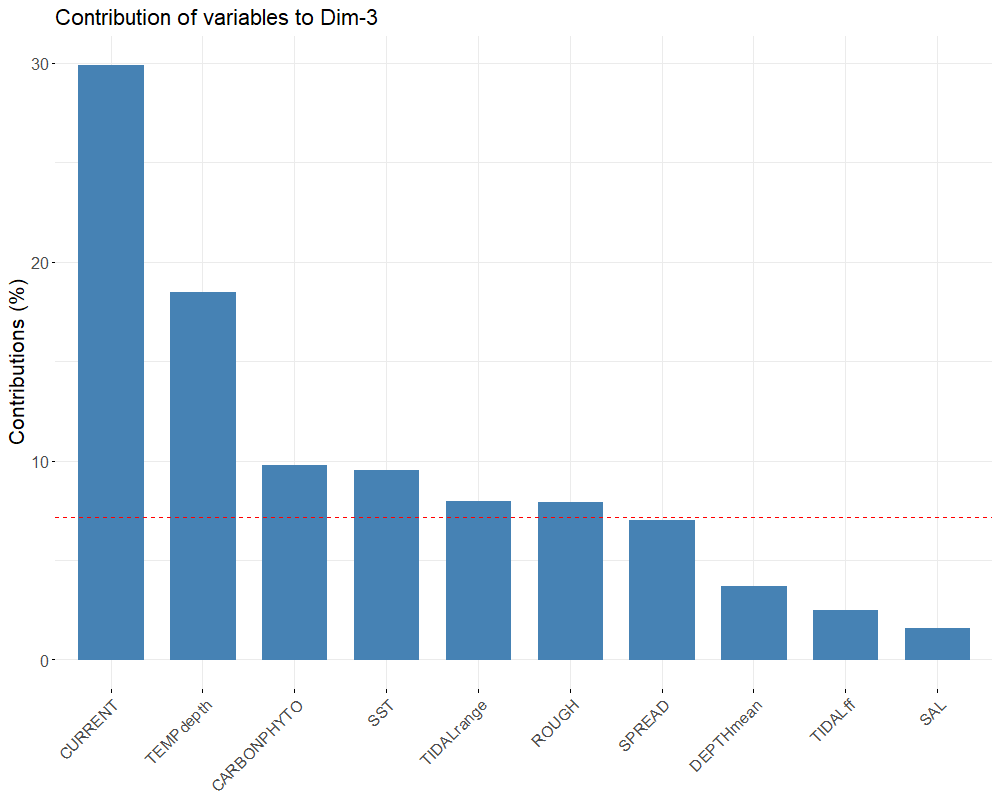


**Fig. S4.4: Plot outlining the relative contributions of different variables to the third dimension of the PCA (PC3 or Dim3)**. The red line is used as a reference, but contributions above 15% were considered the most important in our analysis. Here, current velocity (CURRENT) and temperature at depth (TEMPdepth) meet this criterion. Abbreviations are as provided in **Table S1.1**.


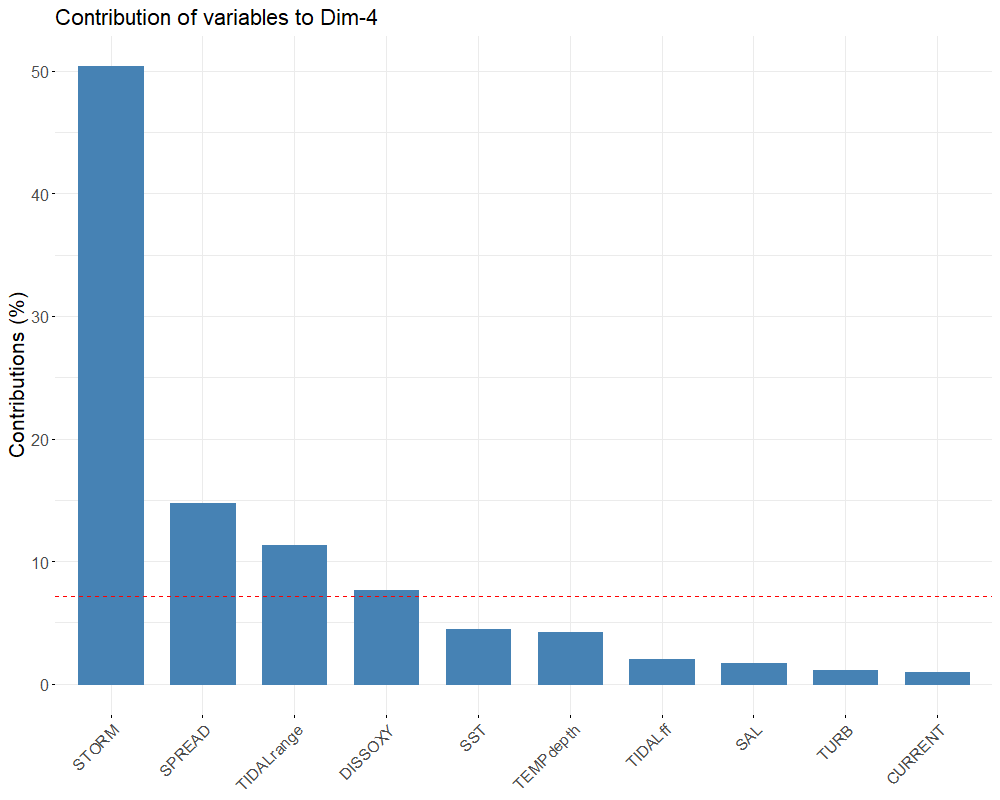


**Fig. S4.5: Plot outlining the relative contributions of different variables to the fourth dimension of the PCA (PC4 or Dim4)**. The red line is used as a reference, but contributions above 15% were considered the most important in our analysis. Here, storm intensity (STORM) meets this criterion. Abbreviations are as provided in **Table S1.1**.


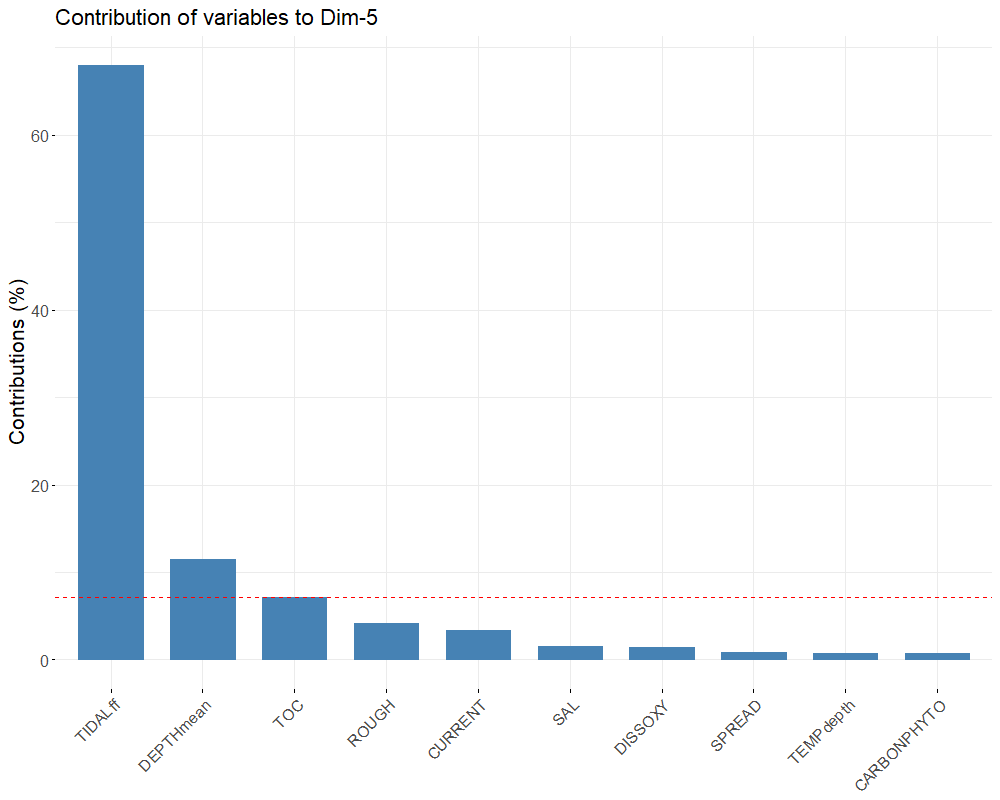


**Fig. S4.6: Plot outlining the relative contributions of different variables to the fifth dimension of the PCA (PC5 or Dim5)**. The red line is used as a reference, but contributions above 15% were considered the most important in our analysis. Here, tidal form factor (TIDALff) meets this criterion. Abbreviations are as provided in **Table S1.1**.

**Table S4.1: Variables explaining the majority of variance on principal component analysis (PCA) components 1-14.** The principal components (PCs) which are not coloured grey are the PCs we considered, explaining most of the variance. For further details and plots of the variable contributions, see accompanying R script and PCA outputs. Abbreviations are as provided in **Table S1.1**.

| Principal Component (PC) | Proportion of variance explained | First three variables, ranked in order of contribution to variance (greatest contribution to least), abbreviated as in **Table S1.1** |
| --- | --- | --- |
| **PC1** | **27%** | **SAL, DISSOXY, SPREAD** |
| **PC2** | **20%** | **TIDALrange, TURB, SST** |
| **PC3** | **13%** | **CURRENT, TEMPdepth, CARBONPHYTO** |
| **PC4** | **9%** | **STORM, SPREAD, TIDALrange** |
| **PC5** | **7%** | **TIDALff, DEPTHmean, TOC** |
| **PC6** | **6%** | **CURRENT, ROUGH, TOC** |
| **PC7** | **5%** | **TEMPdepth, TURB, STORM** |
| **PC8** | **5%** | **CARBONPHYTO, DEPTHmean, CURRENT** |
| **PC9** | **3%** | **TOC, CURRENT, TIDALrange** |
| **PC10** | **2%** | **ROUGH, DEPTHmean, SAL** |
| **PC11** | **1%** | **SPREAD, STORM, ROUGH** |
| **PC12** | **1%** | **TIDALrange, SST, SAL** |
| **PC13** | **<1%** | **TURB, TEMPdepth, SAL** |
| **PC14** | **<1%** | **DISSOXY, SAL, TOC** |


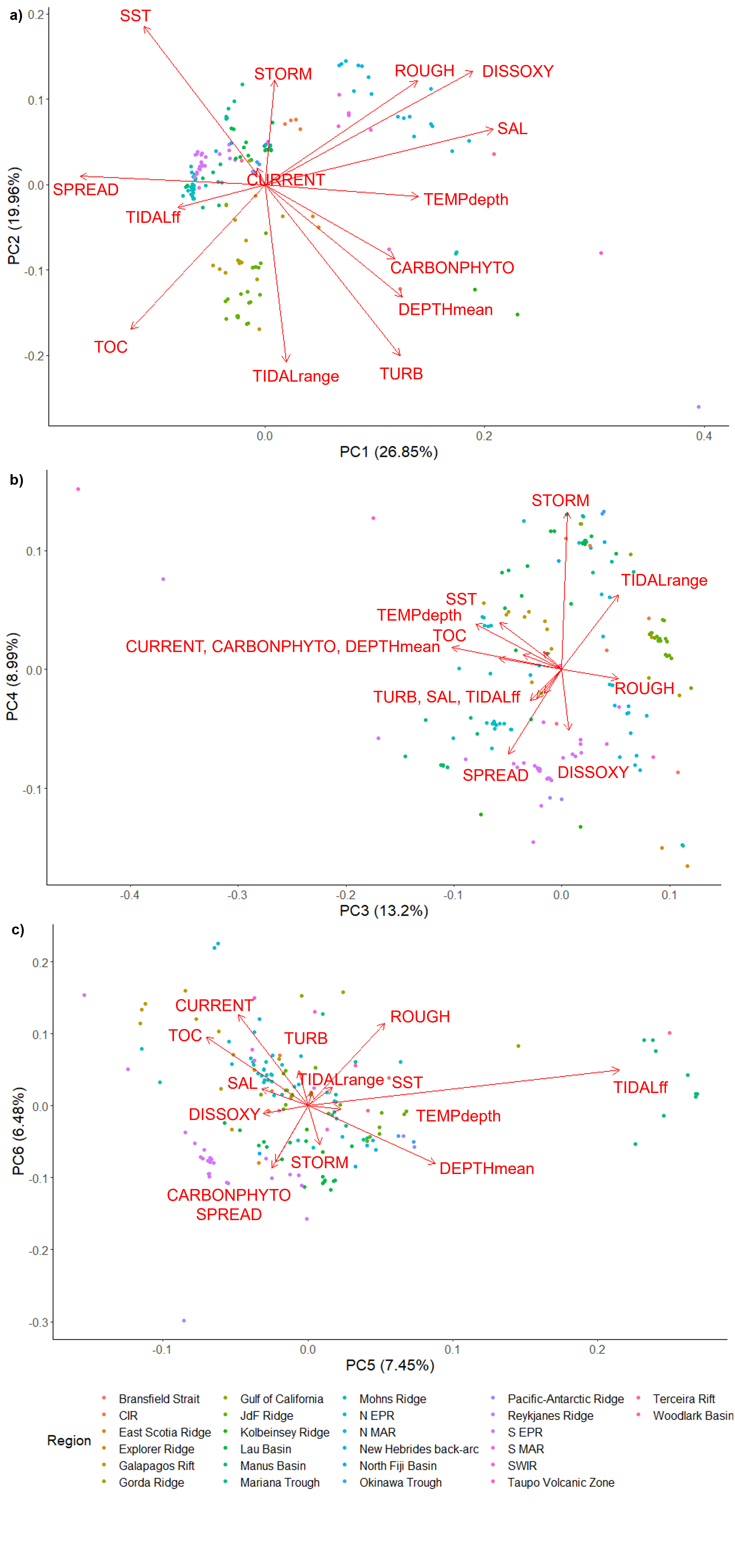


**Fig S4.7: Principal component analysis (PCA) used to identify potential drivers of environmental similarity among 166 vent fields from 14 environmental variables, colour coded by region, to supplement Fig. 3.** Principal Components (PCs) 1-5 capture 76% of the total variance, with 100% explained in 14 PCs. Panel a) shows the first two components of this PCA, together explaining 46.8% of the total variance. Panel b) represents the 3^rd^ and 4^th^ components of this PCA, capturing a further 22.2% of the total variance. Panel c) represents the 5^th^ and 6^th^ components, together explaining a further 13.9% of the total variance. Each point represents a vent field (labelled in **Fig. S1.4**, for reference). Points are coloured according to the region the vent field is found within (according to established vent region names from vent biogeographic literature). Red arrows represent the environmental variables influencing the clustering of fields, with the length of each arrow corresponding to the strength of influence (e.g., on PCs 1 and 2, average depth and phytoplankton in carbon have a similar level of influence, while currents have a much less strong influence than sea surface temperature for some fields). The arrows are labelled according to the abbreviations in **Table S1.1**.

**S5: Supplementary partitioning around medoids (PAM) clustering results**

**Fig. S5.1: (Below and overleaf) Partitioning around medoids (PAM) cluster output**. These are the first two dimensions of the clustering, explaining 57.7% of the variance. Panel a) is an unlabelled plot, where each point represents a vent field. Panel b) includes region labels (which correspond to the regions shown on **Fig. S1.2** panel l) and panel c) includes vent field labels. These labels overlap, so the raw data are best referred to for cluster information for each vent field, but we provide these panels as they highlight vent fields and regions at the edges of clusters, for instance.


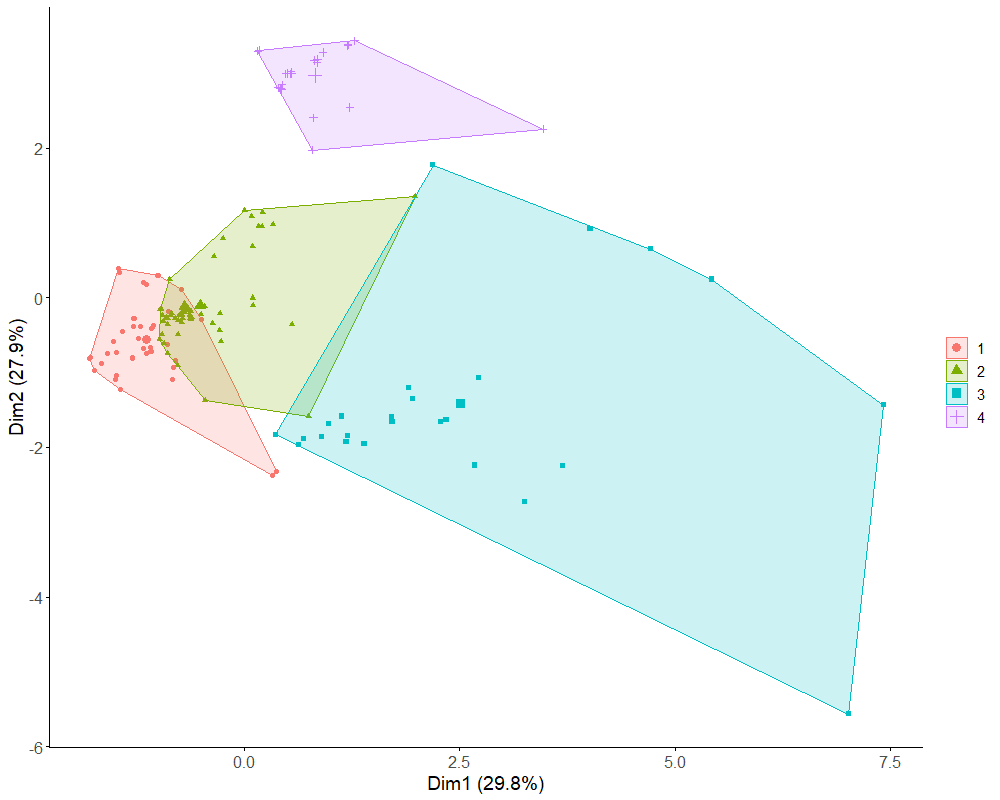


**a)**


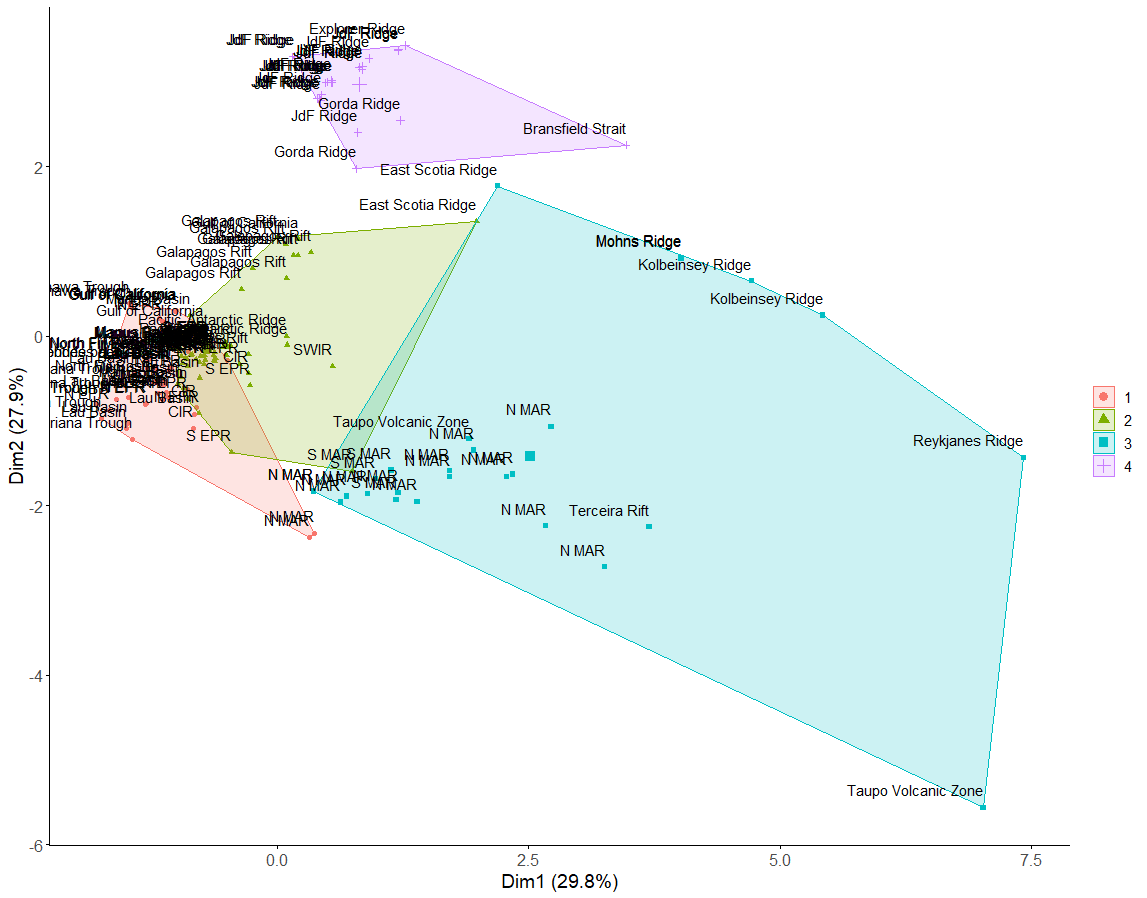


**b)**


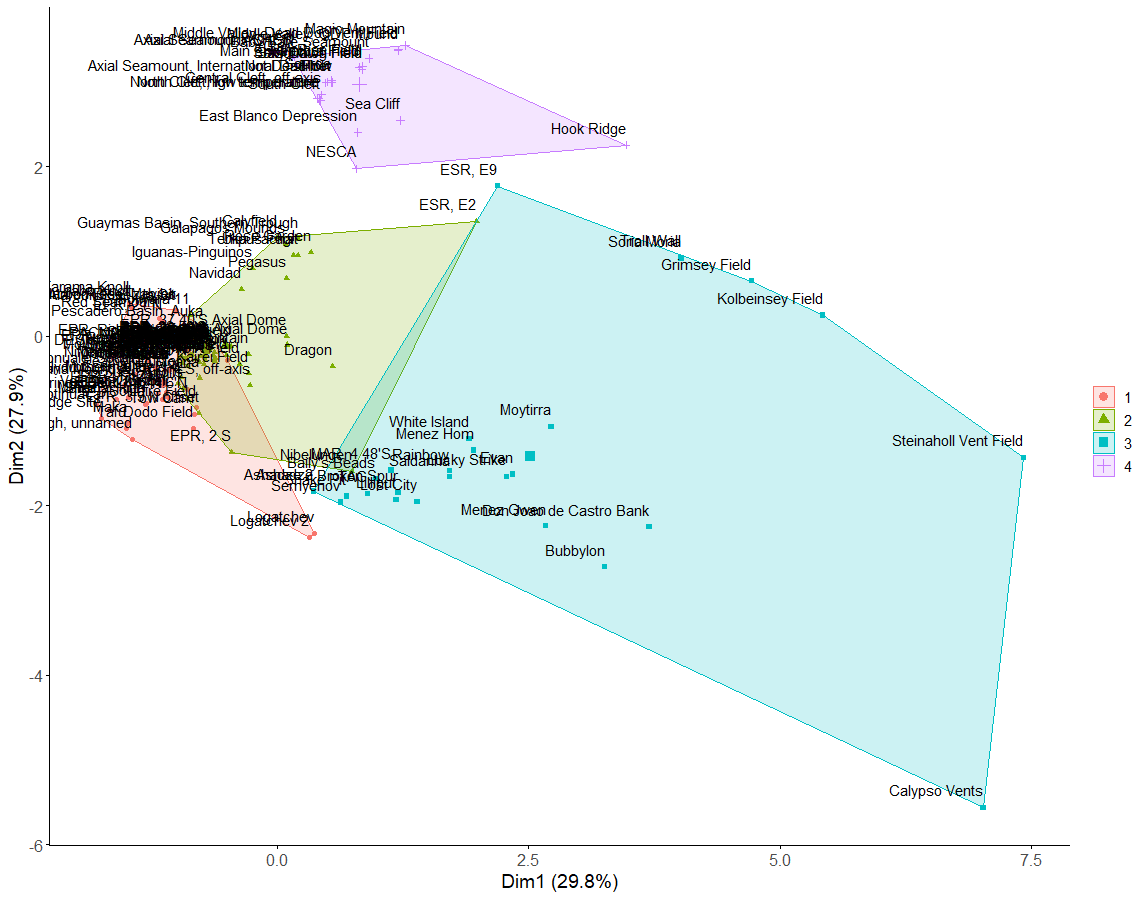


**c)**


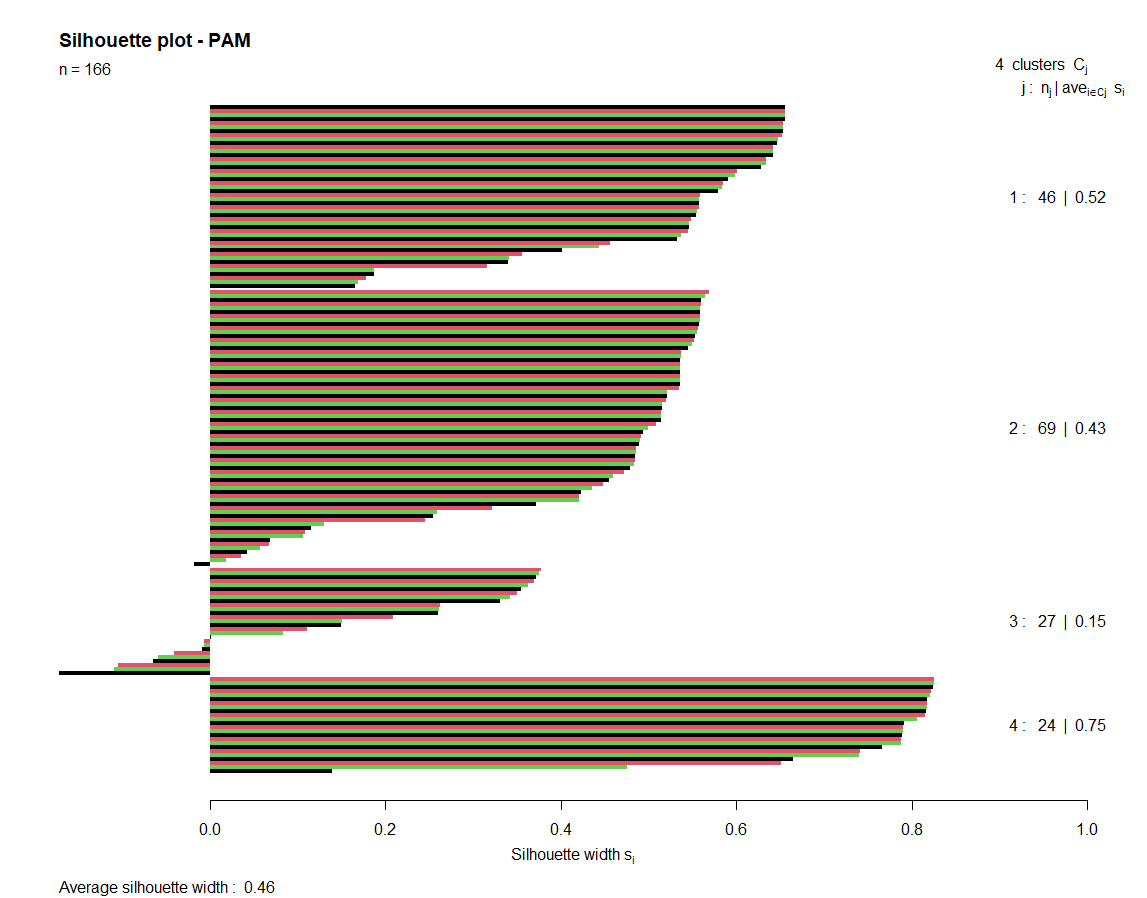


**Figure S5.2: Silhouette plot for PAM clustering with 4 coherent clusters.** Negative silhouette widths suggest less confidence in the cluster assignments. Eleven vent fields have this uncertain cluster assignment, as follows: EPR, 10 44.6'N, Feather Duster, Pegasus, Uka Pacha, Kairei Field, Calyfield, Rose Garden, Tempus Fugit, Galapagos Mounds, Kilo Moana, and CLSC, A3.

**Dataset S5.1:** The data file ‘pam_silwidths_and_clusters.xlsx’ is provided on figshare (<https://doi.org/10.6084/m9.figshare.31558687>) in support of the PAM clustering, giving the silhouette widths associated with each vent field in each cluster. Negative silhouette widths imply less confidence in the cluster assignments, so neighbouring clusters might also be appropriate for fields with negative width values. The dataset has been colour-coded to match the PAM cluster colours presented in the manuscript and supporting figures.

**S6: Manuscript Dataset and R script**

The data and R script to accompany this manuscript are available as supporting files (“environmental_data_all.csv” and “Manuscript_R_Script.R”) at <https://doi.org/10.6084/m9.figshare.31558687> and <https://github.com/abbiesachapman/vent_seascapes>. As some variables were excluded from our analyses, as documented in **Methods**, but could be useful for future research, we include the data for all variables in the Dataset S6 Excel file.

**SUPPLEMENTARY INFORMATION REFERENCES**

1 Beaulieu, S. E. & Szafrański, K. M. InterRidge Global Database of Active Submarine Hydrothermal Vent Fields Version 3.4, doi: 10.1594/PANGAEA.917894 (PANGAEA, 2020).

2 Tyberghein, L. *et al.* Bio-ORACLE: a global environmental dataset for marine species distribution modelling. *Global Ecology and Biogeography* **21**, 272-281, doi:https://doi.org/10.1111/j.1466-8238.2011.00656.x (2012).

3 Assis, J. *et al.* Bio-ORACLE v2.0: Extending marine data layers for bioclimatic modelling. *Global Ecology and Biogeography* **27**, 277-284, doi:https://doi.org/10.1111/geb.12693 (2018).

4 German, C. R., Ramirez-Llodra, E., Baker, M. C. & Tyler, P. A. Deep-water chemosynthetic ecosystem research during the census of marine life decade and beyond: a proposed deep-ocean road map. *PLoS One* **6**, e23259, doi:10.1371/journal.pone.0023259 (2011).

5 UNEP, DEWA & GRID-EUROPE. Tropical Cyclones Windspeed Buffers 1970-2015 (2015).

6 Haigh, I. D. in *Encyclopedia of Maritime and Offshore Engineering* 1-13 (2017).

7 Whittaker, J. M., Goncharov, A., Williams, S. E., Müller, R. D. & Leitchenkov, G. Global sediment thickness data set updated for the Australian-Antarctic Southern Ocean. *Geochemistry, Geophysics, Geosystems* **14**, 3297-3305, doi:https://doi.org/10.1002/ggge.20181 (2013).

8 Amante, C. & Eakins, B. W. ETOPO1 Global Relief Model converted to PanMap layer format, doi: 10.1594/PANGAEA.769615 (PANGAEA, 2009).
